# Users’ preferences and perceptions of the comprehensibility and readability of medication labels

**DOI:** 10.1371/journal.pone.0212173

**Published:** 2019-02-22

**Authors:** Emilia da Silva Pons, Cassia Garcia Moraes, Maicon Falavigna, Lisana Reginini Sirtori, Fernanda da Cruz, Guilherme Webster, Tatiane da Silva Dal Pizzol

**Affiliations:** 1 Proadi-SUS Research Projects Office, Hospital Moinhos de Vento (HMV), Porto Alegre, RS, Brazil; 2 GGREG–General Management Office for Regulations and Good Regulatory Practices, Brazilian Health Regulatory Agency (ANVISA), Brasília, DF, Brazil; 3 General Management Office for Health Inspection and Surveillance, ANVISA, Brasília, DF, Brazil; 4 Independent Graphic Designer, Universidade Federal do Rio Grande do Sul (UFRGS), Porto Alegre, RS, Brazil; 5 Department of Production and Control of Medicines, School of Pharmacy, UFRGS, Porto Alegre, RS, Brazil; University of Milano-Bicocca, ITALY

## Abstract

**Objective:**

To evaluate the labeling preferences of medication users and characterize their perceptions of the comprehensibility and readability of medication labels.

**Methods:**

We conducted a population-based cross-sectional study of medication users aged 18 years or older in 10 Brazilian capital cities. Perceptions of the comprehensibility and readability of medication labels in relation to sociodemographic characteristics were evaluated by Poisson regression models with robust variance. Labeling preferences were assessed through questions addressing possible improvements and through the use of digitally simulated packages.

**Results:**

Of 6,255 medication users interviewed, more than half found it difficult or very difficult to read (50.8%) and/or understand (52.0%) medication labels. Difficulties were more pronounced for participants aged 40 years or older, with lower levels of education, and non-whites. Increasing the font size (93.7%), describing the indications for use (95.9%) and contraindications (95.6%) on the label, and highlighting the expiration date (96.3%) were the most widely accepted improvements. In the evaluation of simulated packages, users preferred factors that improved readability, such as increased font size, use of graphic elements and color to highlight the concentration of the active ingredient, and contrast between the font color and background. The new simulated package design, with increased font size, color to highlight the concentration and contrast between the font color and background, was preferred over the standard design by 77.0% of participants.

**Conclusion:**

Based on users’ perceptions, increased font size and use of graphic elements and color to emphasize critical information, such as expiration date and concentration, are factors that contribute to making medication labels clearer to users. Pharmaceutical industries and policy makers should consider these items when developing labels and defining policies on this issue.

## Introduction

In 1999, the Institute of Medicine published the “To Err is Human” report, estimating 100 000 deaths per year caused by errors in hospital care in the United States [1]. More recent studies estimate that between 210 000 and 440 000 patients each year, in American hospitals, suffer some type of preventable harm that contributes to their death [2]. Medication errors are a major component of errors occurring in care settings. These errors are not limited to the hospital setting, being also considered an important public health problem that mainly affects the vulnerable patient population [3, 4]. Globally, the cost associated with medication errors has been estimated at US$ 42 billion annually [5].

Factors related to medication labeling account for approximately 33% of all medication errors [1]. The label is often the first point of interaction between the user and the medication. Therefore, label information must be legible and understandable to users so that the expected treatment outcomes can be achieved and medication errors can be prevented [6]. Factors such as small font size and style, inadequate spacing between words, and font color without contrasting background may affect both the readability and comprehensibility of medication labels by users, impairing the proper identification of the key information necessary to ensure the safe use of medications. Another factor that hinders the proper identification of medications is look-alike labeling, particularly in generic drugs, where there appears to be a tendency to standardize the layout of drug packages and labels across all product lines from the same manufacturer [6]. Different medications in look-alike packages may increase the risk of confusion between two or more medications by users [6–8]. In this respect, incorporating users’ perception into the package and label design process is essential for the development of readable and understandable products that can ultimately prevent potential medication errors associated with this factor [9–11].

The consequences of medication labeling problems are a concern of different regulatory agencies worldwide. Additionally, users are important stakeholders in the design of medication labels and should be engaged in the process and have their perceptions considered in policy making on this topic.

The objective of the present study was to evaluate the labeling preferences of medication users and characterize their perceptions of the comprehensibility and readability of medication labels.

## Methods

We conducted a cross-sectional study, through a household survey, in 10 Brazilian capital cities (2 capital cities in each of the 5 macro regions of Brazil): Belém, Boa Vista, Cuiabá, Curitiba, Fortaleza, Goiânia, Porto Alegre, Recife, Rio de Janeiro, and São Paulo. Brazil is divided into 5 macro regions (South, Southeast, Midwest, North, and Northeast), consisting of a total of 26 States of the Federation and one Federal District.

This study was commissioned by The Brazilian Health Regulatory Agency (ANVISA) and conducted by Hospital Moinhos de Vento (HMV), a tertiary center of excellence and member facility of a federal government program (PROADI-SUS) that funds the development of research, education, and management activities with potential to contribute to the development of the National Unified Health System (SUS). ANVISA technicians, HMV investigators, and Universidade Federal do Rio Grande do Sul (UFRGS) faculty were involved in the conception and planning of all stages of the study.

The field research team comprised a centralized coordination and an operational support crew, and a team of interviewers. The coordination function was in charge of supervising all data collection processes and stages, including daily quality control of the collected data, while the operational support crew carried out field supervision and provided logistical support to the interviewers. A total of 40 interviewers were trained over the course of a face-to-face session lasting 2 days. The second day of the session was dedicated to pilot testing of the study protocol.

In all 10 capital cities, an equal number of census tracts was randomly selected, according to the 2010 National Census of the Brazilian Institute of Geography and Statistics (IBGE), to obtain a total of 100 census tracts in the whole sample. In each census tract, households were systematically surveyed, respecting an interval of 3 houses, in order to obtain a minimum of 60 residents interviewed. The target population for the survey was adults aged 18 years or older who could read and write and had used at least one medication during the previous year. Individuals who had vision problems but were not wearing glasses or contact lenses at the time of the interview were excluded.

Data were collected, in face-to-face interviews, using electronic devices. Interviews were carried out simultaneously in all 10 selected capital cities; in each city, four interviewers covered the 10 randomly selected census tracts. The data collection instrument consisted of questions addressing the following topics: sociodemographic and economic data; information on medication use; overall perception of difficulty in reading and understanding medication labels currently available in Brazil; overall satisfaction with medication labels; and labeling preferences. For the purposes of this study, label was defined as ‘all information printed on medication packages’.

The following sociodemographic and economic variables were collected: age group (18 to 24 years; 25 to 39; 40 to 59; 60 years or more); schooling (elementary school; high school; college); personal income (no income; up to 1 minimum wage; 1 to 5 times the minimum wage; 5 or more times the minimum wage; did not answer); and self-reported race/ethnicity (white, black, mixed, Asian, indigenous, or did not answer). The variable ‘source of medication supply’ was collected through the question ‘where do you usually obtain your medications?’; responses were coded into the following categories as appropriate: ‘government’ (SUS facilities or Brazilian Popular Pharmacy Program) or ‘commercial’ (retail pharmacies and drugstores).

Overall satisfaction with medication labels available in Brazil was rated on a scale from 0 to 10. Overall perception of difficulty in reading medication labels (readability) was assessed using a 3-point Likert scale for the question ‘Most of the times, how difficult is it for you to read information written on packages?’ Also, overall perception of difficulty in understanding medication labels (comprehensibility) was assessed using a 3-point Likert scale for the question ‘How difficult is it for you to understand information written on packages?’. The 3 points of the Likert scale for both questions were ‘very difficult’, ‘difficult’, and ‘not difficult’.

In order to explore and better understand the factors associated with difficulty in reading and understanding medication labels, two Poisson regression models with robust variance were constructed. Dependent variables in each model were ‘difficulty in reading labels’ (readability) and ‘difficulty in understanding labels’ (comprehensibility). These two dichotomous variables were derived from the answers to the questions described earlier. ‘Very difficult’ and ‘difficult’ answers were categorized as ‘yes’ and ‘not difficult’ answers were categorized as ‘no’ for the variables ‘difficulty in reading labels’ and ‘difficulty in understanding labels’. The following independent variables were tested in each model: gender; age group; schooling; self-reported race/ethnicity; current health problems; and occasional or continued use of medications. The variable ‘current health problems’ was collected through the question ‘Do you have any health condition?’ (yes; no) to provide a measure of respondents’ general state of health. The variable ‘occasional or continued use of medications’ was collected through the question ‘Are you currently taking any medicine?’ (yes; no) and was administered to the whole sample.

Labeling preferences were assessed through questions addressing possible improvements and through the use of digitally simulated packages. The following improvements were proposed to participants: decreased manufacturer name or logo size; less colorful package; addition of color or drawings to package to differentiate between medications; use of color to highlight dose; increased font size; medication contraindications printed on package; indications for use printed on package; and expiration date printed in increased font size and black type.

**Fig 1:**
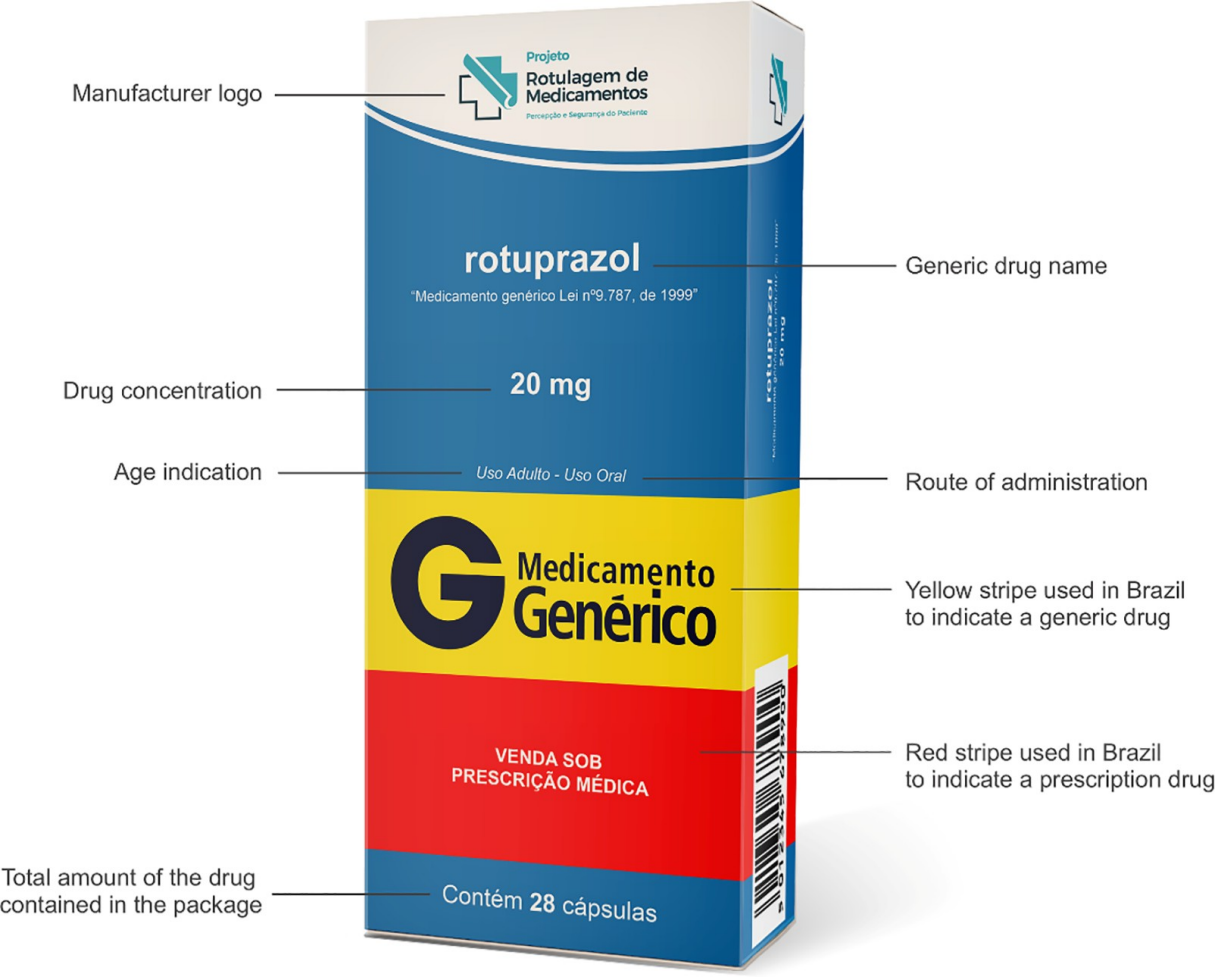
Simulated version of an original package currently marketed in Brazil.

The simulated packages were designed based on internationally accepted guidelines and current Brazilian legislation on medication labeling [6, 12–17]. In order to assess participants’ preferences, each simulated package containing a proposed improvement was compared with a simulated version of an original package currently marketed in Brazil in relation to one of the following aspects: minimum font size; highlighting of different doses of the same medication; vertical or horizontal orientation of text on the label; background color; expiration date and batch number print; use of color to differentiate between drug classes; and use of non-reflective material for blister packs and printing of information on each blister pocket. The layout of the package used as a basis for comparison was identical to that of the medication sold by the leading generic drug manufacturer in Brazil (Fig 1). Two final simulated packages were designed taking into account the aspects proposed individually in the previous simulations. These 2 proposed designs summarize the recommendations for designing safe labels for patients provided in guidelines from the European Union, United Kingdom, Canada, and Australia [6, 12–16].

**Fig 2:**
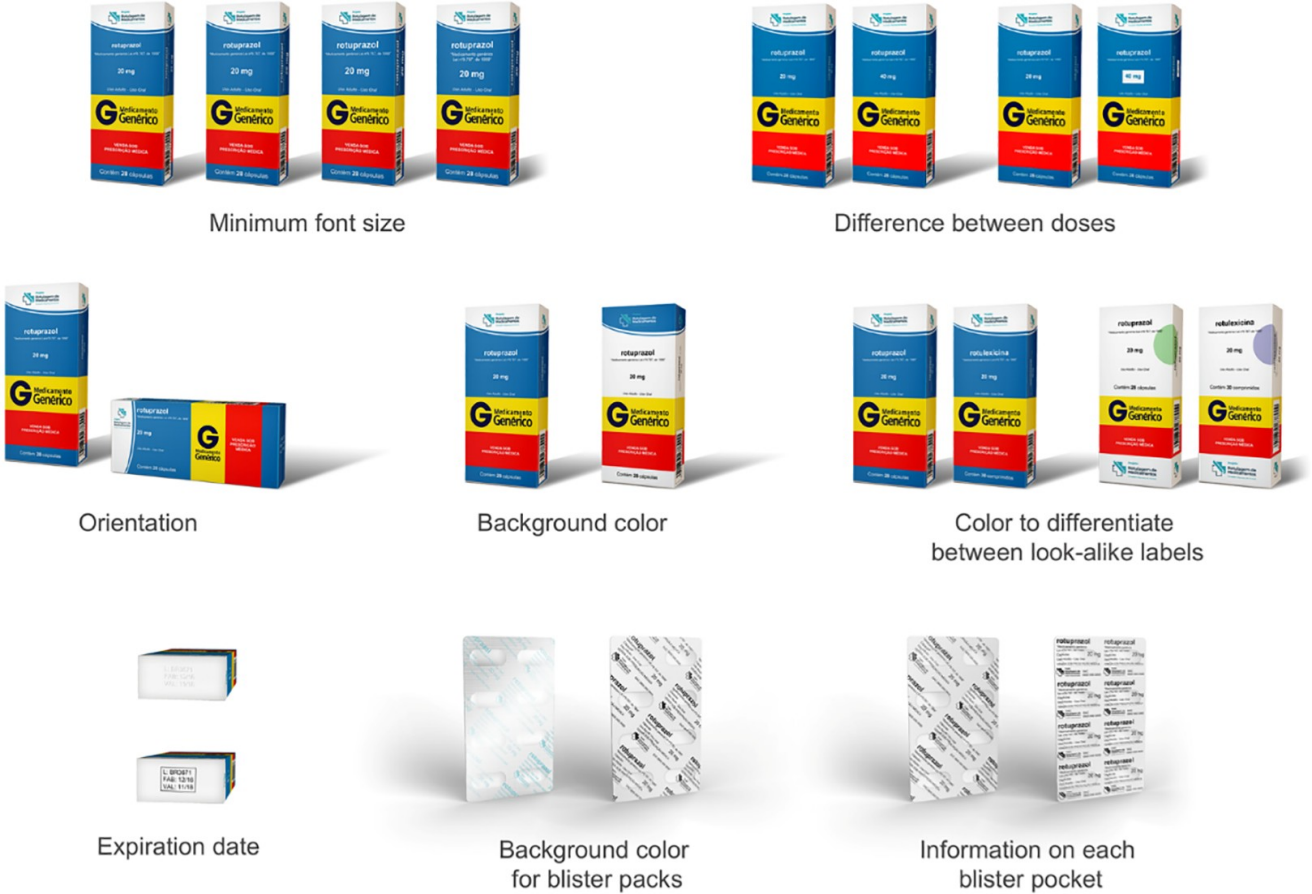
Simulated primary and secondary medication packages presented to participants.

**Fig 3:**
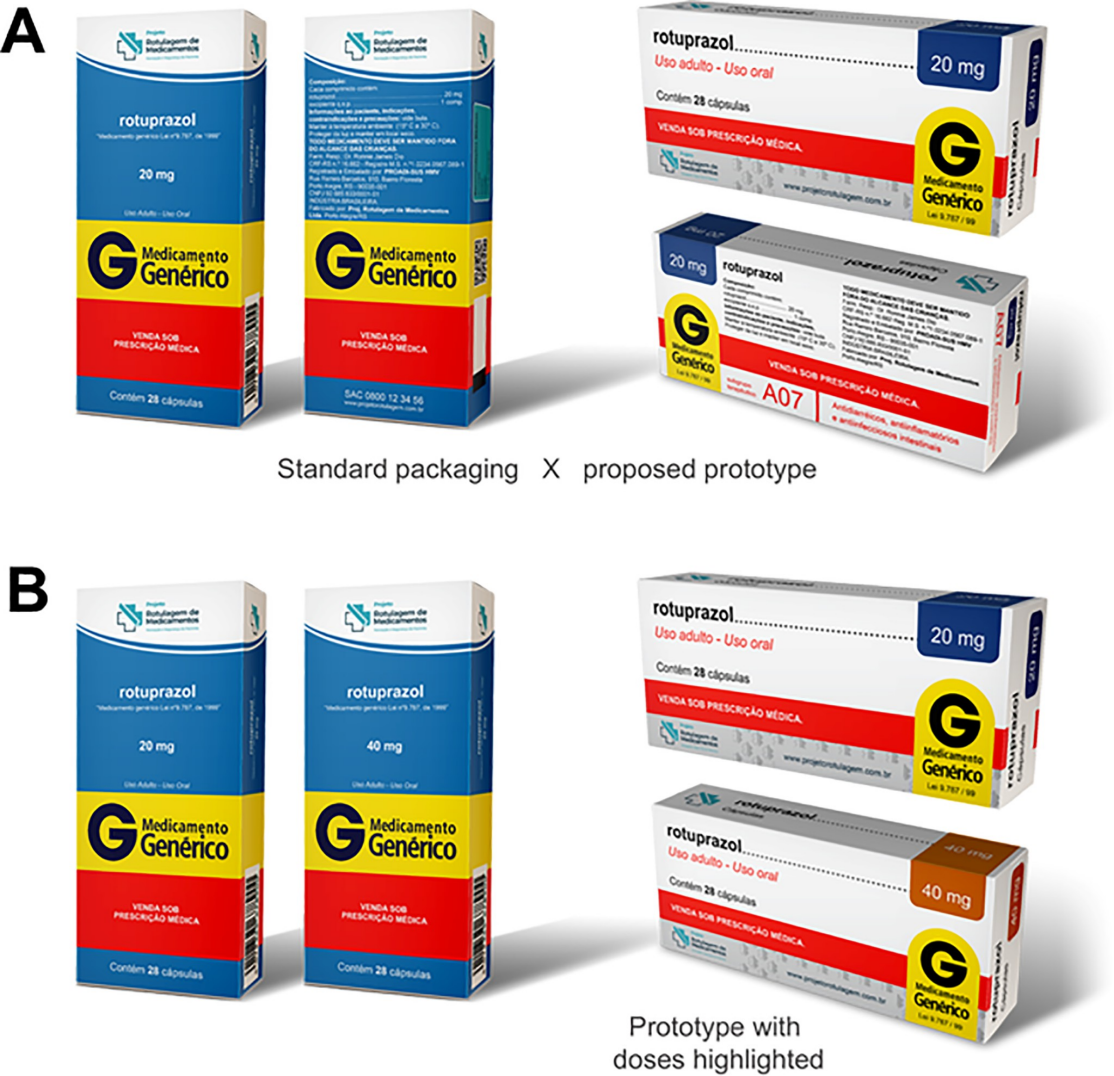
Simulated final proposed designs. A) Standard packaging × proposed prototype. B) Prototype with doses highlighted.

In order to make the interview more dynamic and to prevent the same respondent from evaluating all simulated packages (total 13), we developed 4 different questionnaires. Each questionnaire consisted of 2 different simulations addressing the individual aspects of interest (containing two or three different simulated packages, Fig 2) and 2 final simulations with the proposed improvements applied to the packages (Fig 3). The 2 final simulations were evaluated by the total sample of respondents, while each individual aspect of interest was evaluated by ¼ of the sample. Four interviewers worked in each of the selected cities; each interviewer was randomly assigned one of the 4 different questionnaires to be administered to respondents in their assigned census tracts.

The data collected from the interviews were analyzed using IBM SPSS Statistics for Windows, version 18.0, and expressed as absolute and relative frequencies. Pearson’s chi-square test was used to assess differences between the categories of sociodemographic variables in relation to difficulty in reading and understanding information provided on the medication label. The significance level was set at 5% for all analyses. In the Poisson regression models, the first step in constructing the model was to analyze the variables individually. Those variables that showed statistical significance, defined as p < 0.2, were included in the multivariable model (age group, schooling, self-reported race/ethnicity, current health problems, occasional or continued use of medications). Variables with a statistical significance greater than 0.05 in this step were removed one by one from the model until only those with statistical significance less than 0.05, as determined by the Wald test, remained.

The study was approved by the Research Ethics Committee of Hospital Moinhos de Vento, approval number 1.885.498. Written informed consent was obtained from all participants.

## Results

A total of 6,255 medication users were interviewed in the 10 capital cities sampled from August to September 2017. The sociodemographic characteristics of participants and their information on medication use are shown in Table 1. More than half of the participants were women (54%), 43.9% had completed only elementary school and 43.3% were using at least one medication at the time of the interview.

**Table 1:** Sociodemographic characteristics and information on medication use of the participants in the medication labeling survey. (n = 6,255).

Variable	n	%
Gender		
Male	2875	46.0
Female	3380	54.0
Age group		
18 to 24 years	1082	17.3
25 to 39 years	2190	35.0
40 to 59 years	2039	32.6
60 years or more	944	15.1
Schooling		
Elementary school	2748	43.9
High school	2115	33.8
College	1392	22.3
Personal income^a^		
No income	1423	22.7
Up to 1 minimum wage	2022	32.3
1 to 5 minimum wages	2304	36.8
5 or more minimum wages	258	4.1
Did not answer	248	4.0
Self-reported race/ethnicity		
White	2241	35.8
Black	965	15.4
Mixed	2818	45.1
Asian	111	1.8
Indigenous	71	1.1
Did not answer	49	0.8
Current health problems		
Yes	2296	36.7
No	3959	63.3
Occasional or continued use of medications		
Yes	2706	43.3
No	3549	56.7
Source of medication supply^b^		
Government	3357	42.5
Commercial	4548	57.5
Difficulty in reading medication labels		
Very difficult	696	11.2
Difficult	2473	39.6
Not difficult	3073	49.2
Difficulty in understanding medication labels		
Very difficult	702	11.3
Difficult	2543	40.8
Not difficult	2994	48.0

^a^ In 2017, a minimum wage in Brazil was equivalent to US$ 291.39.

^b^ Multiple response (n = 7,905).

More than half of the participants found it difficult or very difficult to read (50.8%) and understand (52.0%) medication labels. Nearly two-thirds (63.7%) rated their overall satisfaction with medication labels as 7 or less (on a scale from 0 to 10).

Table 2 shows prevalence rates and crude and adjusted prevalence ratios (PR) for the outcomes ‘difficulty in reading labels’ and ‘difficulty in understanding labels’ according to sociodemographic characteristics, current health problems, and occasional or continued use of medications. Statistically significant differences (p < 0.001) were found in users’ perceptions of their difficulty in reading and understanding labels in relation to age, schooling, and race. Difficulties were particularly pronounced for participants aged 40 years or older, with lower levels of education, and non-whites.

**Table 2:** Overall perception of medication users of difficulty in reading and understanding medication labels. (n = 6,255).

Characteristics	Difficulty in reading labels						Difficulty in understanding labels					
	Prevalence (%)	p-value^a^	PRc	p-value^b^	PRa	p-value^b^	Prevalence (%)	p-value^a^	PRc	p-value^b^	PRa	p-value^b^
Gender		0.280		0.649				0.334		0.312		
Female	50.5		1				51.4		1			
Male	51.1		1.01 (0.96–1.06)				52.7		1.02 (0.98–1.07)			
Age Group		<0.001		<0.001		<0.001		<0.001		<0.001		<0.001
18 to 24 years	40.1		1		1		43.4		1		1	
25 to 39 years	43.8		1.09 (1.00–1.19)		1.10 (1.01–1.20)		47.7		1.10 (1.01–1.19)		1.11 (1.02–1.20)	
40 to 59 years	60.8		1.57 (1.40–1.64)		1.50 (1.39–1.63)		58.9		1.35 (1.25–1.46)		1.35 (1.25–1.46)	
60 years or more	57.4		1.43 (1.30–1.57)		1.42 (1.29–1.55)		57.3		1.32 (1.21–1.44)		1.32 (1.20–1.44)	
Schooling		<0.001		<0.001		<0.001		<0.001		<0.001		<0.001
College	41.3		1		1		43.0		1		1	
High school	48.6		1.18 (1.09–1.27)		1.14 (1.06–1.23)		50.8		1.18 (1.10–1.27)		1.15 (1.06–1.23)	
Elementary school	57.2		1.38 (1.29–1.49)		1.27 (1.18–1.37)		57.6		1.34 (1.25–1.44)		1.25 (1.16–1.34)	
Race		<0.001		<0.001		<0.001		<0.001		<0.001		<0.001
White	45.2		1		1		45.3		1		1	
Non-white^c^	53.9		1.19 (1.13–1.26)		1.20 (1.13–1.26)		55.9		1.23 (1.17–1.30)		1.23 (1.16–1.30)	
Current health problems		<0.001		<0.001				<0.001		<0.001		
No	47.7		1				49.6		1			
Yes	56.0		1.17 (1.12–1.23)				56.2		1.13 (1.08–1.19)			
Occasional or continued use of medications		<0.001		<0.001				<0.001		<0.001		
No	48.4		1				50.1		1			
Yes	53.9		1.11 (1.06–1.17)				54.6		1.09 (1.04–1.14)			

^a^ p-values were calculated using Pearson’s chi-square test.

^b^ p-values were calculated using Poisson regression models with robust variance.

^c^ Black, Mixed, Asian and Indigenous

PR_c,_ Crude prevalence ratio; PR_a,_ Adjusted prevalence ratio

**Fig 4:**
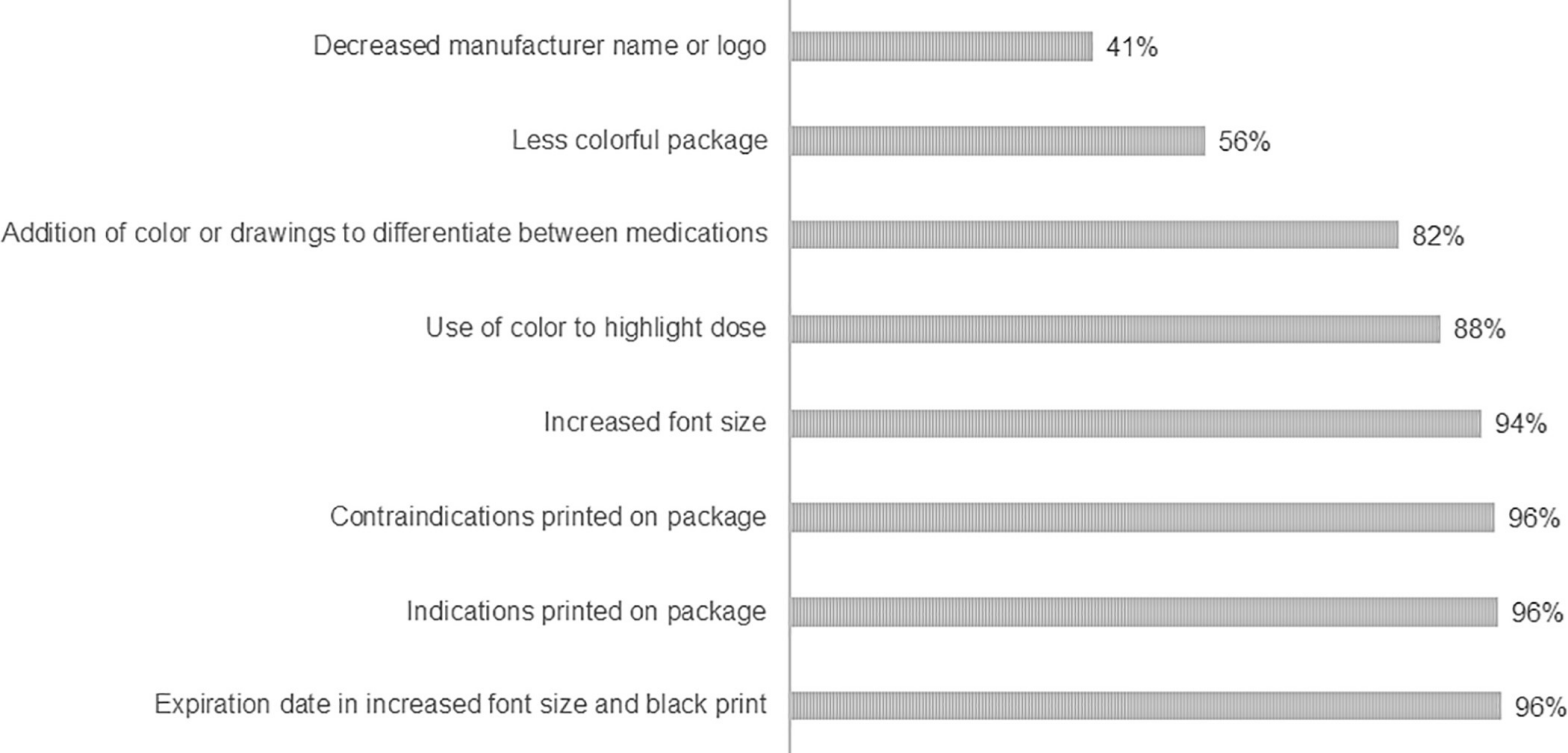
Users’ acceptance of possible label improvements. (n = 6225).

Users’ acceptance of possible label improvements is shown in Fig 4. Of all improvements proposed to users, increasing the font size (93.7%), describing the indications (95.9%) and contraindications (95.6%) on the label, and highlighting the expiration date (96.3%) were the most widely accepted improvements.

Table 3 shows users’ labeling preferences obtained through the evaluation of simulated packages (Fig 2). Of particular note is the almost unanimous preference of users for printing of expiration date with contrast between the font color and background over embossing or debossing of text onto the package (98.6%) and for using blister packs with a white background over uncoated aluminum foil (98.1%). The preference rate for the final proposed design (Fig 3A) was 77.0% compared with the simulated package with a layout identical to that of the medication sold by the leading generic drug manufacturer in Brazil. Interestingly, participants preferred the use of vertical text on the package when this aspect was evaluated individually, although in the most widely accepted final prototype, among other aspects, the text was printed horizontally.

**Table 3:** Medication users’ labeling preferences obtained through the evaluation of simulated packages. (n = 6,225^a^).

Secondary package–Drug cartons		Primary package–Blister packs	
Labeling aspects	Preference (%)	Labeling aspects	Preference (%)
Minimum font size for drug name		Background color	
Arial 7.5 point	20.0	Aluminum foil	1.1
Arial 11 point	13.9	White	98.1
Arial 14 point	18.1	Indifferent	0.8
Arial 16 point	48.0	Information on each blister pocket	
Difference between doses		Yes	95.6
Not highlighted	7.9	No	2.1
Highlighted	83.2	Indifferent	2.3
Indifferent	8.9	Expiration date	
Orientation of text		Engraving (embossing or debossing)	1.9
Vertical	63.0	Black print	97.6
Horizontal	25.7	Indifferent	0.5
Indifferent	11.3	Prototypes^a^	
Background color		Standard	18.1
Blue	37.9	Proposed	77.0
White	52.4	Indifferent	4.9
Indifferent	9.7	Prototypes with dose highlighted ^a^	
Expiration date		Standard	16.2
Engraving (embossing or debossing)	0.3	Proposed	80.1
Black print	98.6	Indifferent	3.7
Indifferent	1.1		
Color to differentiate between drug classes			
Yes	68.0		
No	28.0		
Indifferent	4.0		

^a^ The 2 final simulations were evaluated by the total sample of respondents, while each individual aspect of interest was evaluated by ¼ of the sample.

## Discussion

More than half of the interviewed Brazilian medication users found it difficult to read and understand medication labels available in Brazil. This finding is a warning sign, since the difficulties encountered by users may lead to incorrect and unsafe use of medications, jeopardizing patient safety and imposing an economic burden on the health system [18–20].

Safe medication identification, selection and administration depend not only on the user’s ability to read and understand information on labels but also on features inherent in labeling, such as typographic style and design aspects [21, 22]. In our study, 63.7% of participants reported low satisfaction with medication labels.

For the interviewed users, increased font size (93.7%) and use of contrasting background to highlight the expiration date (96.3%) and dose (88.0%) are measures that may contribute to improving labels. Studies have shown that small font size, look-alike labeling and absence of contrast between the font color and background for information on dose and expiration date are factors that impair the readability of labels [6, 10, 23, 24]. Readability issues due to small font size, for example, can make it difficult to read important information required to correctly use the medication, such as concentration, route of administration, and storage conditions. Look-alike labeling, in turn, a common issue mainly in generic drugs, can contribute to medication mix-ups, which appear in our study as one of the probable reasons for users’ dissatisfaction, since the suggestion to add color or drawings to differentiate between look-alike labels was accepted by 82.4% of participants.

Another strategy used in our study to assess users’ opinions about possible label improvements was the analysis of simulated packages. The simulation results confirm the importance of factors such as increased font size, emphasis on the concentration of the active ingredient, and use of contrasting background to highlight the expiration date. When participants were presented with a type style in four point sizes and asked to choose the smallest font size with which they felt most comfortable to read information provided on the label, the largest font size (Arial 16 point) was preferred twice as much as the other three options (Arial 7.5, 11, and 14 point). In the study conducted by Smither and Braun, the 14-point prints were considered easier to read than the 9- and 12-point prints [25]. Wogalter and Vigilante evaluated consumers’ preference and understanding of labels with three different font sizes: 4, 7, and 10 point [23]. They concluded that larger font size is preferred and better understood by older adults, while, for younger adults, font size has no influence on preference and understanding of labels. Current Brazilian legislation does not establish the minimum font size to be used on labels. In international guidelines, however, the minimum recommended font size is 12 point [6, 13, 17].

The use of graphic elements or color to highlight the concentration of the active ingredient helped distinguish between different concentrations of the same medication for 83.2% of participants. Studies have shown that the use of color (as opposed to monochrome) for key information, such as medication name and concentration, helps users identify information faster and more accurately [24, 26, 27]. Guidelines for the design of medication labels recommend the use of graphic elements, such as different type styles, boldface, colors, and shapes, to highlight concentrations mainly on look-alike packages from the same manufacturer [6, 13, 17]. In Brazil, current legislation on medication labeling does not address the use of graphic elements or color to highlight the concentration of the active ingredient [16].

Batch number and expiration date are essential information for product traceability and safe medication use. Although national legislation prohibits the embossing or debossing of batch number and expiration date onto a package when this method does not provide sufficient contrast on its own, this is a common practice in Brazil [16]. The identification of this information is hindered when the text is embossed or debossed without contrast between the text and the background [13]. Regarding users’ preference, expiration date printed in black type on a white contrasting background on packages and on blister packs was preferred over debossing of text without color by 98.6% and 97.6% of participants, respectively.

On blister packs, the readability of information can be affected by the type of material used. Reflective foils, such as uncoated aluminum foil, reduce the visibility of information, especially when such information is printed in a light color on a low-contrast color background [13]. In the present study, simulated blister packs with an uncoated aluminum foil surface as the printing surface were strongly rejected by the participants. Ideally, information should be printed on non-reflective matt foils or colored foils [6, 13].

Information printed on blister packs is essential for the correct identification of the medication by the user, especially when the outer package and accompanying package inserts are discarded. In this respect, information printed on blister packs must remain available until the last unit dose is removed [6, 12, 13]. Therefore, the information should be repeated on each blister pocket in order to improve the readability of key information and allow the correct identification of the medication [6, 12, 13]. The importance of this recommendation was recognized by the interviewed users, since 95.6% of them preferred the information repeated on each blister pocket over the information printed across the entire blister strip, where the text can be damaged when the medication is removed.

According to users’ opinions, indications for use and contraindications should also appear on the label. More than 90% of participants found it important that information on what the medication is used for and who cannot take the medication be displayed on the label. From the users’ point of view, some studies indicate that, in addition to information that helps identify the medication, the indications, dose, dosage, and associated risks are viewed as the most important information [28–31]. The importance of such information for most respondents may suggest that users are interested in obtaining information because they perceive a need to deal with issues related to their health. In Brazil, the description of therapeutic indications on the label is mandatory only for over-the-counter medications.

Based on the results of the present study, there is a clear need for improvement in medication labels. Most improvements proposed to users were widely accepted when tested individually. The final simulated packages, which combined the individually tested improvements, were preferred by 77% (Fig 3A) and 80.9% (Fig 3B) of participants, confirming the low level of satisfaction of users with current labels and a preference for change. In the final simulations, there was a clear preference of users for factors that improved readability, such as increased font size, use of graphic elements and color to highlight the concentration of the active ingredient, and contrast between the font color and background.

We carried out bivariate analyses to check for possible associations between the variables gender, age, and schooling and the two final simulated packages presented to respondents (Fig 3). We found that the horizontal prototypes were preferred by women, by younger respondents, and by those with higher levels of education, although the difference was modest (data not shown). On the other hand, the elderly and the interviwees with lower levels of education preferred the vertical prototypes. It is possible that older respondents and those with less lower level of schooling preferred vertical labels due to greater cognitive difficulty in these groups, and that, regardless of the type of label or packaging, these individuals would experience difficulties in reading and comprehension. Particularly for people with a lower level of schooling or health literacy, medication labels (and package inserts) should be a complement, not a substitute for verbal information provided by health professionals.

Only a few studies have focused on surveying users’ opinions and, mainly, their understanding of medication labels, especially in developing countries. To our knowledge, this is the largest study evaluating users’ understanding, perception, cognition and preferences regarding medication labels, with over 6,000 people interviewed, in a complex sampling process, with cluster sampling and systematic selection of participants. This study characterized users’ perceptions of the readability and comprehensibility of medication labels available in Brazil by interviewing a comprehensive sample of medication users living in capital cities of the 5 macro regions of Brazil. As stated earlier, medication labels tend to show similarities across different countries. Therefore, our findings may be considered generalizable to different contexts, especially to low- and middle-income countries, where the educational and sociocultural level of the population may be closer to that of Brazil than of high-income countries. Of note, Brazil is a continental country with high population variability across different regions, which has a positive impact on the external validity of our findings.

Our study included only state capital cities, with a population ranging from 332 thousand to 12 million inhabitants, which may be considered a limitation of the study. Although the use of simulated packages to evaluate users’ preferences is a differential advantage of this study over other studies on this topic, the simulated packages were presented as 2D images under standard conditions. Therefore, we cannot state that, if physical package prototypes were used, which allow greater interaction between the user and the package, users’ perceptions of the readability and comprehensibility of information would remain the same.

Users’ difficulties in reading and understanding medication labels were evident from the findings of the present study. These difficulties reflect the need for improvements in labels in order to make them clearer and self-explanatory to users. The proper identification of information presented on the label is important for safe medication use, since studies have shown that a portion of medication errors is due to labeling confusion [1, 7]. In this respect, we concluded that increased font size, use of graphic elements, color and contrast, and use of coherent hierarchical levels of information in the design of packages to emphasize critical information, such as concentration and expiration date, are factors that contribute to making medication labels clearer to users. Pharmaceutical industries and policy makers should consider these items when developing labels and defining policies on this issue.

We also identified the importance of involving users, particularly those with greater difficulty in understanding labels (older, less educated, and non-white individuals), in the development of policies and regulatory strategies aimed at medication labeling. Users’ perceptions, preferences and opinions can contribute to the design of safer labels for the population, minimizing medication errors and, consequently, having a positive impact on public health. The strategy of incorporating users’ perception into the development of regulations is a way of bringing developers and users closer together. By recognizing users’ needs, public policies can be shaped within an experimental, evidence-based framework.

In our study, the participants preferred the labels that met international standards, including increased font size and use of graphic elements and color to emphasize critical information, such as expiration date and concentration. Although there is a national standard in Brazil that establishes rules for medication labels [16], this standard has several deficiencies, many of which correspond to the issues identified by the respondents. The findings of this study may help developing countries, such as Brazil, develop or revise their local regulations so that medication labels both meet user preferences and are as close as possible to international (e.g., European Union, United Kingdom, Canada, Australia) standards. Likewise, it may help drug companies that produce medications for developing countries where no official standard may exist. Pharmaceutical industries should apply these principles and involve users in the design of medication labels, focusing on patient safety rather than merely on business strategies.

In conclusion, based on users’ perceptions, increased font size and use of graphic elements and color to emphasize critical information–such as expiration date and concentration ¬–are factors that contribute to making medication labels clearer to users.

## Supporting information

S1 Dataset (XLSX)

S1 Questionnaire (PDF)

S2 Questionnaire (PDF)

S3 Questionnaire (PDF)

S4 Questionnaire (PDF)

S5 Questionnaire (Portuguese). (PDF)

## References

[1] Institute of Medicine (US) Committee on Quality of Health Care in America. To err is human. Washington (DC): National Academies Press (US); 2000 [cited 2018 Apr 04]. Available from: https://www.ncbi.nlm.nih.gov/books/NBK225182/.

[2] James JT. A new, evidence-based estimate of patient harms associated with hospital care. Journal of patient safety. 2013;9(3):122–8. 10.1097/PTS.0b013e3182948a69 . 23860193

[3] Shrank WH, Parker R, Davis T, Pandit AU, Knox JP, Moraras P, et al Rationale and design of a randomized trial to evaluate an evidence-based prescription drug label on actual medication use. Contemporary clinical trials. 2010;31(6):564–71. 10.1016/j.cct.2010.07.004 . 20647058

[4] World Health Organization (WHO). Medication errors: technical series on safer primary care Geneva: WHO; 2016 [cited 2018 Apr 04]. Available from: http://apps.who.int/iris/bitstream/10665/252274/1/9789241511643-eng.pdf.

[5] World Health Organization (WHO). Medication without harm: WHO's third global patient safety challenge Geneva: WHO; 2017 [cited 2018 Apr 04]. Available from: http://apps.who.int/iris/bitstream/10665/255263/1/WHO-HIS-SDS-2017.6-eng.pdf?ua=1&ua=1.

[6] Health Canada. Good label and package practices guide for prescription drugs Ottawa: Health Canada; 2016 [cited 2018 Apr 04]. Available from: https://www.canada.ca/content/dam/hc-sc/migration/hc-sc/dhp-mps/alt_formats/pdf/pubs/medeff/guide/2016-label-package-practices-pratiques-etiquetage-emballage-rx/glppg-gbpee-rx-eng.pdf.

[7] Berman A. Reducing medication errors through naming, labeling, and packaging. Journal of medical systems. 2004;28(1):9–29. . 15171066 10.1023/b:joms.0000021518.60670.10

[8] Institute for Safe Medication Practices Canada (ISPM). Labelling and packaging: an aggregate analysis of medication incident reports Toronto: Institute for Safe Medication Practices Canada 2013 [cited 2018 Apr 04]. Available from: https://www.ismp-canada.org/download/LabellingPackaging/ISMPC2013_LabellingPackaging_FullReport.pdf.

[9] Cho J, Miller SR, Simpson TW, Shooter SB. Effects of over-the-counter medication product family packaging design on knowledge acquisition and consumer preferences. ASME Proceedings 26th International Conference on Design Theory and Methodology. 2014;V007T07A039.

[10] Wilke T, Müller S, Neumann K, Loder T. Does package design matter for patients? Pharmaceut Med. 2011;25(5):307–17.

[11] Wolf MS, Davis TC, Shrank W, Rapp DN, Bass PF, Connor UM, et al To err is human: patient misinterpretations of prescription drug label instructions. Patient education and counseling. 2007;67(3):293–300. 10.1016/j.pec.2007.03.024 . 17587533

[12] European commission enterprise and industry directorate-general. Guideline on the readability of the label and package leaflet of medicinal products for human use Brussels: European Commission; 2009 [cited 2018 Apr 04]. Available from: https://ec.europa.eu/health//sites/health/files/files/eudralex/vol-2/c/2009_01_12_readability_guideline_final_en.pdf.

[13] National Patient Safety Agency and the Helen Hamlyn Research Centre. Design for patient safety A guide to the graphic design of medication packaging London: National Health Service; 2007 [cited 2018 Apr 04]. Available from: http://www.hhc.rca.ac.uk/cms/files/npsa-design-for-patient-safety-.pdf.

[14] National Health Service. National Patient Safety Agency Design for patient safety: a guide to the labelling and packaging of injectable medicines London: National Health Service; 2008 [cited 2018 Apr 04]. Available from: http://www.nrls.npsa.nhs.uk/resources/?entryid45=59831.

[15] Australian Government. Department of Health. Therapeutic Goods Administration. Medicine labels: guidance on TGO 91 and TGO 92 Symonston: Department of Health; 2016 [cited 2018 Apr 04]. Available from: https://www.tga.gov.au/sites/default/files/medicine-labels-guidance-tgo-91-and-tgo-92.pdf.

[16] Brasil. Ministério da Saúde. Agência Nacional de Vigilância Sanitária. Resolução-RDC Nº 71, de 22 de dezembro DE 2009. Estabelece regras para a rotulagem de medicamentos. [cited 2018 Apr 04]. Available from: http://bvsms.saude.gov.br/bvs/saudelegis/anvisa/2009/res0071_22_12_2009.html.

[17] U.S. Department of Health and Human Services. Food and Drug Administration. Center for Drug Evaluation and Research (CDER) Safety considerations for product design to minimize medication errors guidance for industry Rockville: Food and Drug Administration; 2016 [cited 2018 Apr 04]. Available from: https://www.fda.gov/downloads/drugs/guidancecomplianceregulatoryinformation/guidances/ucm331810.pdf.

[18] Bohra AS, Tiwari P. An investigational study on the legibility of eye drops’ labels. Indian J Pharm Sci. 2006;68(5):677–9.

[19] Bauer DT, Guerlain S. Improving the usability of intravenous medication labels to support safe medication delivery. International journal of industrial ergonomics. 2011;41(4):394–9. 10.1016/j.ergon.2011.02.005 21731156 PMC3126152

[20] Ward J, Clarkson PJ, Buckle P, Harris W. The packaging and labelling of solid oral medicine using oral methotrexate as an example. ASME Proceedings Medical and Healthcare Engineering. 2004;3:459–68.

[21] Buckle P, Clarkson PJ, Coleman R, Ward J, Anderson J. Patient safety, systems design and ergonomics. Applied ergonomics. 2006;37(4):491–500. 10.1016/j.apergo.2006.04.016 . 16753132

[22] Medicines and Healthcare products Regulatory Agency. Best practice guidance on patient information leaflets 2014 [cited 2018 Apr 04]. Available from: https://www.gov.uk/government/publications/best-practice-guidance-on-patient-information-leaflets.

[23] Wogalter MS, Vigilante WJ Jr. Effects of label format on knowledge acquisition and perceived readability by younger and older adults. Ergonomics. 2003;46(4):327–44. 10.1080/0014013021000048006 . 12637173

[24] Hellier E, Tucker M, Kenny N, Rowntree A, Edworthy J. Merits of using color and shape differentiation to improve the speed and accuracy of drug strength identification on over-the-counter medicines by laypeople. Journal of patient safety. 2010;6(3):158–64. . 21491790 10.1097/pts.0b013e3181eee157

[25] Smither JA, Braun CC. Readability of prescription drug labels by older and younger adults. Journal of clinical psychology in medical settings. 1994;1(2):149–59. 10.1007/BF01999743 . 24227289

[26] Hellier E, Edworthy J, Derbyshire N, Costello A. Considering the impact of medicine label design characteristics on patient safety. Ergonomics. 2006;49(5–6):617–30. 10.1080/00140130600568980 . 16717013

[27] Endestad T, Wortinger LA, Madsen S, Hortemo S. Package Design Affects Accuracy Recognition for Medications. Human factors. 2016;58(8):1206–16. 10.1177/0018720816664824 27591209 PMC5570154

[28] Koo MM, Krass I, Aslani P. Factors influencing consumer use of written drug information. The Annals of pharmacotherapy. 2003;37(2):259–67. 10.1177/106002800303700218 . 12549958

[29] Vigilante WJ, Wogalter MS. The preferred order of over-the-counter (Otc) pharmaceutical label components. Drug Inf J. 1997;31(3):973–88.

[30] Shrank WH, Agnew-Blais J, Choudhry NK, Wolf MS, Kesselheim AS, Avorn J, et al The variability and quality of medication container labels. Archives of internal medicine. 2007;167(16):1760–5. 10.1001/archinte.167.16.1760 . 17846395

[31] Grime J, Blenkinsopp A, Raynor DK, Pollock K, Knapp P. The role and value of written information for patients about individual medicines: a systematic review. Health expectations: an international journal of public participation in health care and health policy. 2007;10(3):286–98. 10.1111/j.1369-7625.2007.00454.x 17678517 PMC5060401

