# Supplementary material for: Users’ preferences and perceptions of the comprehensibility and readability of medication labels
Source: PLoS One. 2019 Feb 22;14(2):e0212173. doi: 10.1371/journal.pone.0212173 (PMC6386266; doi:10.1371/journal.pone.0212173)

QUESTIONNARIE – USERS OF MEDICINES

QUESTIONNARIE 3– DIPYRONE AND CLORANA® (PRIMARY PACKAGE)

In this interview, we will talk about drug labels available in Brazil. Labels are information printed on drug packages. Examples of labels include the information printed on drug cartons and blister packs, and adhesive labels affixed to vials. In this interview, we will not talk about drug package inserts. I will begin by asking you some questions about yourself:

PART 1 – INFORMATION ABOUT THE INTERVIEWEE

	City of interview:<br><br>*write down without asking*	[] Belém<br>[] Boa Vista<br>[] Cuiabá<br>[] Curitiba<br>[] Fortaleza<br>[] Goiânia<br>[] Porto Alegre<br>[] Recife<br>[] Rio de Janeiro<br>[] São Paulo	
	What is your first and last name?		
	Could you give me a phone number?		
	Interviewee sex:<br>**write down without asking**	[] Male<br>[] Female	
	How old are you?		
	Can read and write?	[] Yes<br>[] No	
	What school grade have you completed?<br>**Consider completed school grades**		
	Primary school (first years of elementary school)	[]	1 [] 2 [] 3 [] 4 [] 5 []
	Admission test	[]	4 []
	Middle school or junior high school	[]	1 [] 2 [] 3 [] 4 []
	Complete elementary school either at a regular school or at an adult education program	[]	1 [] 2 [] 3 [] 4 [] 5 [] 6 [] 7 [] 8 [] 9 []

	High school, normal school, or vocational school either at a regular school or at an adult education program	[]	1 [] 2 [] 3 []
	Undergraduate education	[]	[] Complete<br>[] Incomplete
	Graduate education (specialization, master's degree, PhD)	[]	
	Never studied	[]	
	Do not know/Did not answer	[]	
	What is your family monthly income?		
	What is your skin color or race?<br>*Read the options*	[] White<br>[] Black<br>[] Asian<br>[] Mixed<br>[] Native Brazilian<br>[] Do not know/Did not answer	

PART 2 – HEALTH INFORMATION

	Do you have any health condition?	[] Yes<br>[] No
	Are you currently taking any medicine?	[] Yes<br>[] No - **skip the next question**
	How many different medicines are you currently taking?	
	Where do you usually obtain these medicines?<br>*Read the options*<br>*Multiple choice*	- [] Brazilian Unified Health System (SUS)<br>- [] Brazilian Popular Pharmacy Program (Programa Farmácia Popular)<br>- [] Private pharmacy<br>- [] Other<br>- [] Do not know/Did not answer
	Do you obtain your medicines yourself?	[] Yes<br>[] No
	Does anyone help you use your medicines?	[] Yes<br>[] No
	Do you usually help someone use medicines?	[] Yes<br>[] No
	Do you need to wear glasses or contact lenses?	[] Yes<br>[] No - **skip the next question**
	Are you wearing glasses or contact lenses now?<br># If the answer is NO, finish the interview	[] Yes<br>[] No

I will now make some questions about some drug labels. To answer these questions, I will show you some drug packages.

PART 5 – PRIMARY PACKAGE – PRESCRIPTION DRUG

Hand the* clorana® *package to the interviewee, as previously established by draw

UNDERSTANDING – PRIMARY PACKAGE – CLORANA®

Does the label that you see shows that:

	The product is hydrochlorothiazide?<br>**Read the options**	[] True<br>[] False<br>[] Do not know/Did not answer
	Each pill contains 25 mg?<br>**Read the options**	[] True<br>[] False<br>[] Do not know/Did not answer

READABILITY – PRIMARY PACKAGE – CLORANA®

How difficult it is for you to:

	Read the name hydrochlorothiazide on this package?<br>**Read the options**	[] Very difficult<br>[] Difficult<br>[] Not difficult<br>[] Do not know/Did not answer
	Read the number of milligrams on this package?<br>**Read the options**	[] Very difficult<br>[] Difficult<br>[] Not difficult<br>[] Do not know/Did not answer
	Read the expiration date on this package?<br>**Read the options**	[] Very difficult<br>[] Difficult<br>[] Not difficult<br>[] Do not know/Did not answer

PART 6 – PRIMARY PACKAGE (SMALL VIAL)

** Hand the dipyrone package to the interviewee, as established by draw**

UNDERSTANDING – DIPYRONE

Does the label that you see shows that the product:

	Contains dipyrone?<br>**Read the options**	[] True<br>[] False<br>[] Do not know/Did not answer
	Contains 500 mg/mL?<br>**Read the options**	[] True<br>[] False<br>[] Do not know/Did not answer

READABILY – DIPYRONE

How difficult is it for you to:

	Read the name dipyrone on this package?<br>**Read the options**	[] Very difficult<br>[] Difficult<br>[] Not difficult<br>[] Do not know/Did not answer
	Read the number of milligrams on this package?<br>**Read the options**	[] Very difficult<br>[] Difficult<br>[] Not difficult<br>[] Do not know/Did not answer
	Read the expiration date on this package?<br>**Read the options**	[] Very difficult<br>[] Difficult<br>[] Not difficult<br>[] Do not know/Did not answer
	Read information about what the medicine is for?<br>**Read the options**	[] Very difficult<br>[] Difficult<br>[] Not difficult<br>[] Do not know/Did not answer

Take the drug packages back from the interviewee

I will now ask you about your general experience with drug labels available in Brazil.

PART 7 – SATISFACTION

Considering your general experience with medicines:

	How difficult is it for you to **understand** information written on packages?<br>**Read the options**	[] Very difficult<br>[] Difficult<br>[] Not difficult<br>[] Do not know/Did not answer
	Most of the times, how difficult is it for you to **read** information written on packages?<br>**Read the options**	[] Very difficult<br>[] Difficult<br>[] Not difficult<br>[] Do not know/Did not answer
	Do you usually read the address of the company that produced the medicine on the package?<br>**Read the options**	[] Yes<br>[] No<br>[] Do not know/Did not answer
	Do you usually read who is the technical responsible for the medicine on the package?<br>**Read the options**	[] Yes<br>[] No<br>[] Do not know/Did not answer
	Do you usually read the sentence: "ALL MEDICINES SHOULD BE KEPT OUT OF CHILDREN'S REACH."	[] Yes<br>[] No<br>[] Do not know/Did not answer
	In your opinion, is it important that the package inform about what the medicine is used for?<br>**Read the options**	[] Yes<br>[] No<br>[] Do not know/Did not answer
	In your opinion, is it important that the package inform about who should not take the medicine?<br>**Read the options**	[] Yes<br>[] No<br>[] Do not know/Did not answer
	In your opinion, what should contribute to improve medicine labels:	
	Increasing the font size?	[] Yes<br>[] No<br>[] Do not know/Did not answer
	Highlighting the number of milligrams with colors?	[] Yes<br>[] No<br>[] Do not know/Did not answer
	Writing the expiration date with a larger font size and in black font?	[] Yes<br>[] No<br>[] Do not know/Did not answer
	Using less colorful packages?	[] Yes<br>[] No<br>[] Do not know/Did not answer
	Reducing the size of company's logo?	[] Yes<br>[] No<br>[] Do not know/Did not answer

	Using colors or draws to differentiate what each medicine is used for?	[] Yes<br>[] No<br>[] Do not know/Did not answer
	On a scale from 1 to 10, how satisfied are you with drug labels available in Brazil?	

I will now show you some pictures of drug labels and make some questions about them.

**PART 8 – PROTOTYPES**

	**PROTOTYPE 3** – In which of these two packages can drug information be more easily read?	[] Package A (original)<br>[] Package B (prototype)<br>[] Indifferent<br>[] Do not know/Did not answer
	**PROTOTYPE 7** – In which of these pairs do you think is easier to identify that they are two different medicines?	[] Pair of Packages A (original)<br>[] Pair of Packages B (prototype)<br>[] Indifferent<br>[] Do not know/Did not answer
	**PROTOTYPE 10** – In which of these packages is it easier to read the drug name?	[] Pair of Packages A (original)<br>[] Pair of Packages B (prototype)<br>[] Indifferent<br>[] Do not know/Did not answer
	**PROTOTYPE 12** – In which of these pairs of packages is it easier to visualize important information for those taking the medicine?	[] Pair of Packages A (original)<br>[] Pair of Packages B (prototype)<br>[] Indifferent<br>[] Do not know/Did not answer
	**PROTOTYPE 13** – In which of these pairs of packages is it easier to visualize important information for those taking the medicine?	[] Pair of Packages A (original)<br>[] Pair of Packages B (prototype)<br>[] Indifferent<br>[] Do not know/Did not answer

PROTOTYPES USED IN THE INTERVIEWS

PROTOTYPE 3 – Orientation of Text Information on Drug Carton

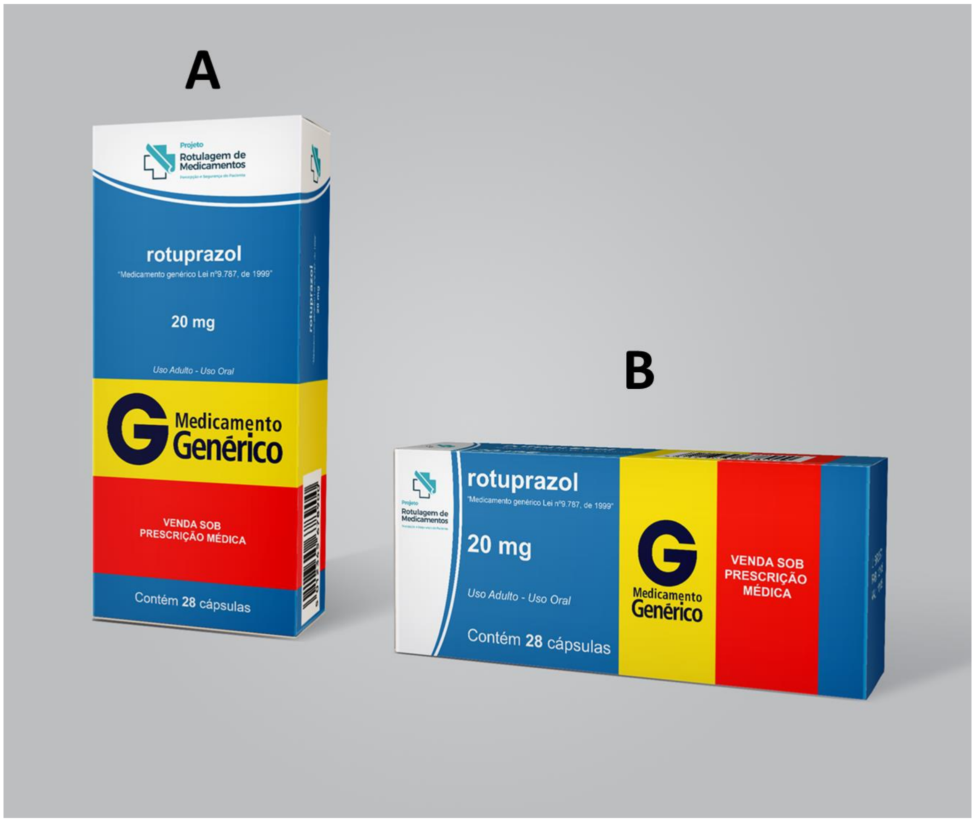

PROTOTYPE 7 - Colors to Identify Therapeutic Class

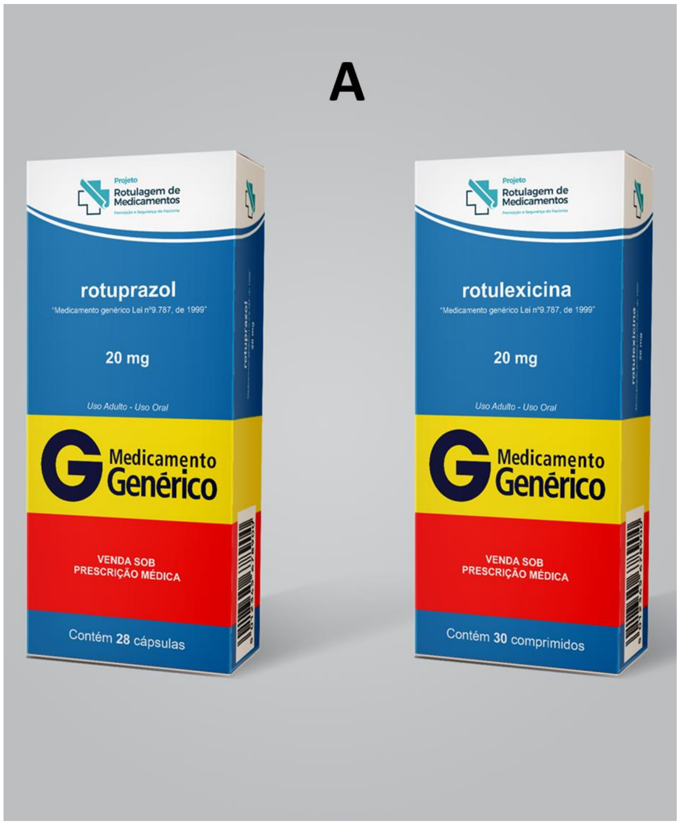

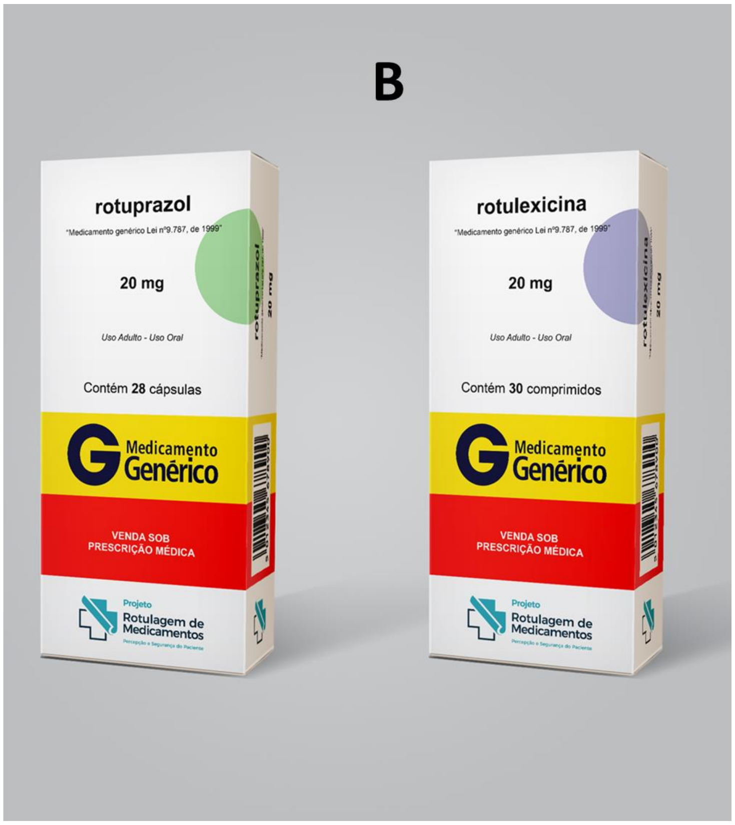

PROTOTYPE 10 – Blister Pack for Over-the-Counter Drugs

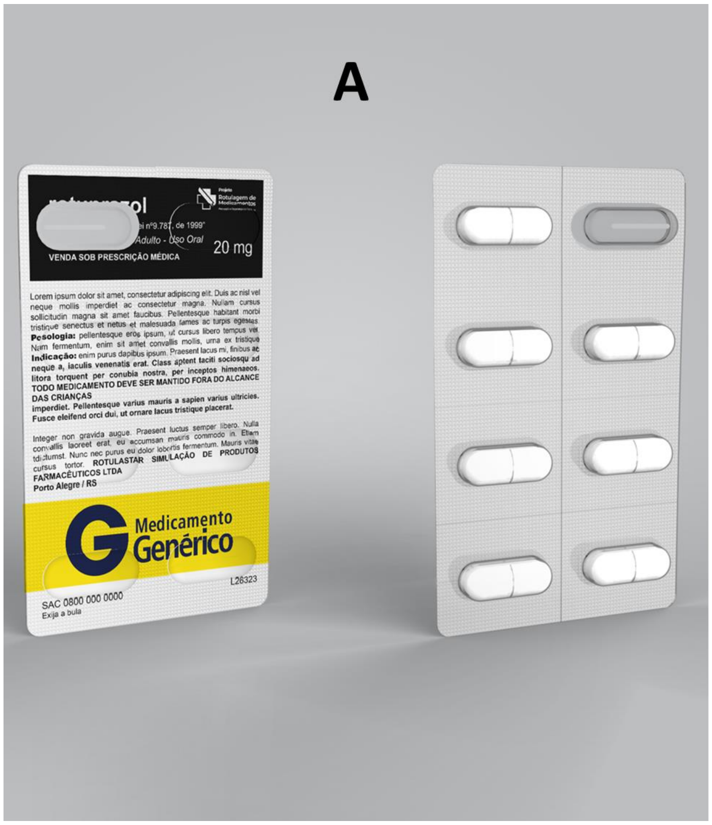

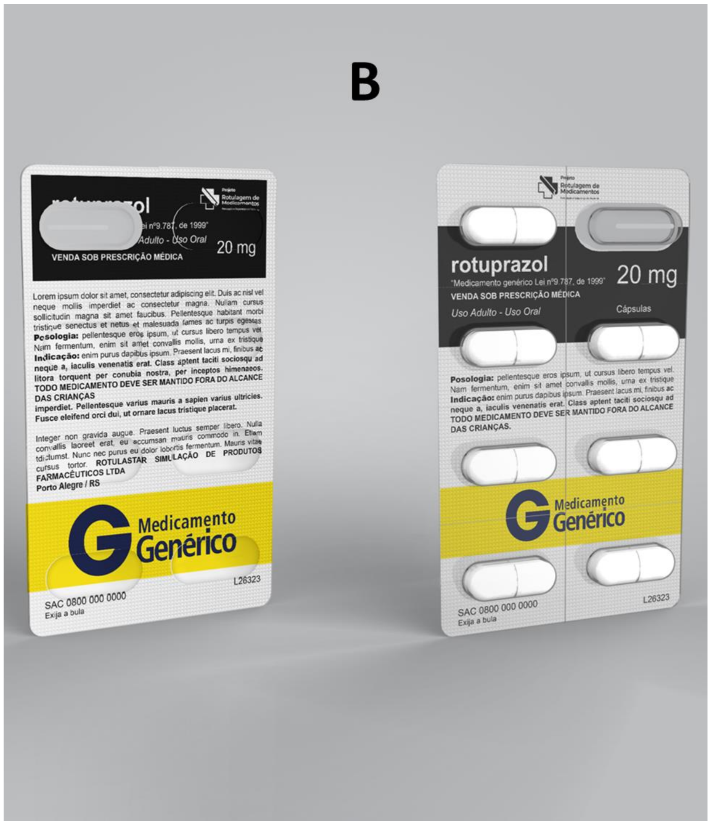

PROTOTYPE 12 – Ideal Package

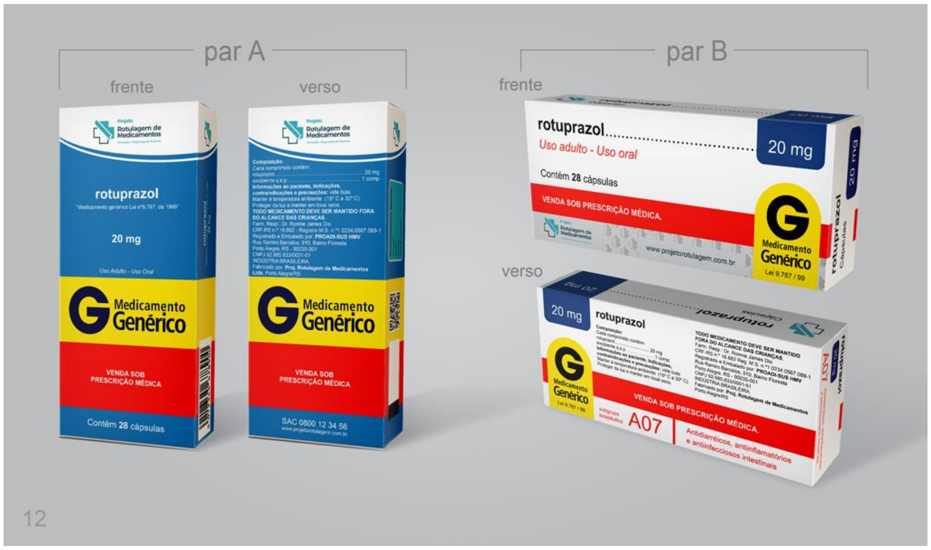

PROTOTYPE 13 – Ideal Package with Highlighted Doses

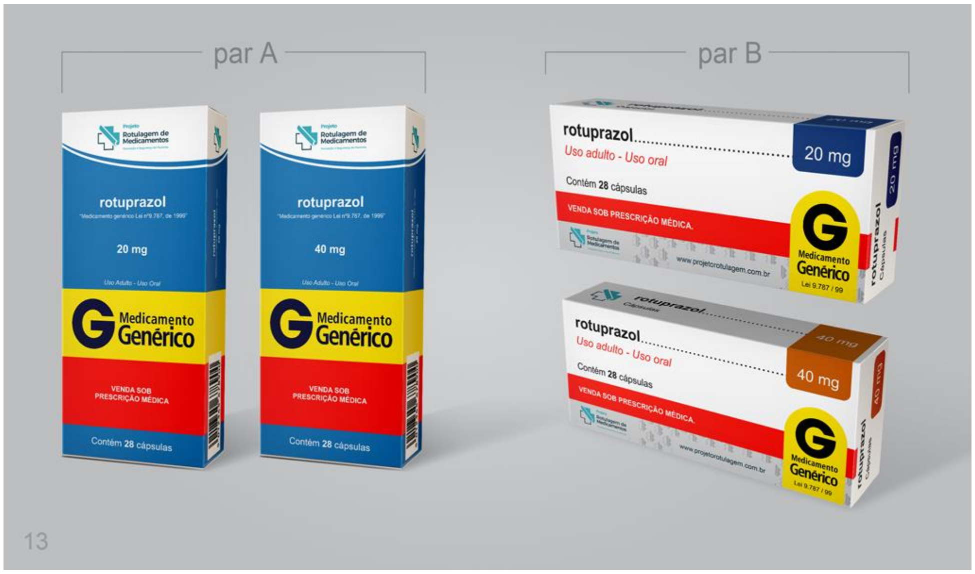

Supplement: S3 Questionnaire — (PDF) [file pone.0212173.s004.pdf]
