# Supplementary material for: Users’ preferences and perceptions of the comprehensibility and readability of medication labels
Source: PLoS One. 2019 Feb 22;14(2):e0212173. doi: 10.1371/journal.pone.0212173 (PMC6386266; doi:10.1371/journal.pone.0212173)

Hospital Moinhos de Vento | Afiliado a Johns Hopkins Medicine International | PROADI-SUS Programa de Apoio ao Desenvolvimento Institucional do Sistema Único de Saúde

Projeto
Rotulagem de Medicamentos

QUESTIONÁRIO USUÁRIOS DE MEDICAMENTOS

Nesta entrevista vamos conversar sobre os rótulos dos remédios disponíveis no Brasil. Os rótulos são todas as informações impressas nas embalagens dos remédios. Como exemplo, são rótulos as informações impressas nas caixas dos remédios e nas cartelas, e os adesivos colados nos frascos. Nesta entrevista não vamos falar sobre as bulas. Vou começar esta entrevista fazendo algumas perguntas sobre você:

BLOCO 1 – INFORMAÇÕES SOBRE O ENTREVISTADO

	Cidade onde está realizando a entrevista:<br><br>*anotar sem perguntar*	[] Belém<br>[] Boa Vista<br>[] Cuiabá<br>[] Curitiba<br>[] Fortaleza<br>[] Goiânia<br>[] Porto Alegre<br>[] Recife<br>[] Rio de Janeiro<br>[] São Paulo	
	Qual é o seu nome e último sobrenome?		
	Você poderia me informar um número de telefone?		
	Sexo do entrevistado:<br>**anotar sem perguntar**	[] Masculino<br>[] Feminino	
	Qual é a sua idade?		
	Você sabe ler e escrever?	[] Sim<br>[] Não	
	Até que série e grau você estudou?<br>**Considere as séries completas de estudo**		
	Curso primário	[]	1 [] 2 [] 3 [] 4 [] 5 []
	Admissão	[]	4 []
	Curso ginasial ou ginásio	[]	1 [] 2 [] 3 [] 4 []
	1º grau ou fundamental ou supletivo de primeiro grau ou EJA	[]	1 [] 2 [] 3 [] 4 [] 5 [] 6 [] 7 [] 8 [] 9 []
	2º grau ou colégio técnico ou normal ou científico ou ensino médio ou supletivo de segundo grau ou EJA	[]	1 [] 2 [] 3 []
	3º grau ou curso superior	[]	[] Completo

			[] Incompleto
	Pós- graduação (especialização, mestrado, doutorado)	[]	
	Nunca estudou	[]	
	NS/NR	[]	
	Qual a sua renda familiar mensal?		
	A sua cor ou raça é:<br>*Ler as alternativas*	[] Branca<br>[] Preta<br>[] Amarela<br>[] Parda<br>[] Indígena<br>[] NS/NR	

BLOCO 2 – INFORMAÇÕES DE SAÚDE

	Você tem algum problema de saúde?	[] Sim<br>[] Não
	No momento, você está tomando algum remédio?	[] Sim<br>[] Não - **pula**
	Quantos remédios diferentes você está tomando no momento?	
	Onde você costuma adquirir seus remédios?<br>*Ler as alternativas*<br>*Escolha múltipla*	- [] SUS<br>- [] Programa Farmácia Popular<br>- [] Farmácia particular<br>- [] Outro<br>- [] NS/NR
	É você mesmo quem adquire os remédios que usa?	[] Sim<br>[] Não
	Alguém lhe ajuda a usar os seus remédios?	[] Sim<br>[] Não
	Você costuma ajudar alguém a usar algum remédio?	[] Sim<br>[] Não
	Você precisa usar óculos ou lentes de contato?	[] Sim<br>[] Não – **pula**
	Você está usando os óculos ou lentes de contato nesse momento?<br># Se a resposta for NÃO, encerrar a entrevista	[] Sim<br>[] Não

Agora vou lhe fazer algumas perguntas sobre os rótulos de alguns remédios. Para responder a estas perguntas, eu lhe mostrarei algumas embalagens de remédios.

BLOCO 3 – EMBALAGEM PRIMÁRIA

*Dar a embalagem de Paracetamol ou Tylenol® para o entrevistado, conforme sorteio *

COMPREENSÃO – PARACETAMOL ou TYLENOL®

O rótulo que você está vendo indica que o produto:

	É paracetamol<br>*Ler as alternativas*	[] Verdadeiro<br>[] Falso<br>[] NS/NR
	Cada comprimido contém 750 mg do remédio<br>*Ler as alternativas*	[] Verdadeiro<br>[] Falso<br>[] NS/NR

LEGIBILIDADE – PARACETAMOL ou TYLENOL®

O quão difícil é para você:

	Ler o nome paracetamol nesta embalagem?<br>*Ler as alternativas*	[] Muito difícil<br>[] Difícil<br>[] Não é difícil<br>[] NS/NR
	Ler os miligramas nesta embalagem?<br>*Ler as alternativas*	[] Muito difícil<br>[] Difícil<br>[] Não é difícil<br>[] NS/NR
	Ler a validade nesta embalagem?<br>*Ler as alternativas*	[] Muito difícil<br>[] Difícil<br>[] Não é difícil<br>[] NS/NR
	Ler para o que serve esse remédio?<br>*Ler as alternativas*	[] Muito difícil<br>[] Difícil<br>[] Não é difícil<br>[] NS/NR

BLOCO 4 – EMBALAGEM SECUNDÁRIA – MEDICAMENTO DE VENDA SOB PRESCRIÇÃO MÉDICA

Dar a embalagem de Omeprazol ou Losec® para o entrevistado, conforme sorteio

COMPREENSÃO – EMBALAGEM SECUNDÁRIA – OMEPRAZOL OU LOSEC®

O rótulo que você está vendo indica que o produto:

	Tem omeprazol<br>**Ler as alternativas**	[] Verdadeiro<br>[] Falso<br>[] NS/NR
	Cada cápsula contém 40 mg<br>**Ler as alternativas**	[] Verdadeiro<br>[] Falso<br>[] NS/NR

LEGIBILIDADE – EMBALAGEM SECUNDÁRIA – OMEPRAZOL OU LOSEC®

O quão difícil é para você:

	Ler o nome do remédio nesta embalagem?<br>**Ler as alternativas**	[] Muito difícil<br>[] Difícil<br>[] Não é difícil<br>[] NS/NR
	Ler os miligramas nesta embalagem?<br>**Ler as alternativas**	[] Muito difícil<br>[] Difícil<br>[] Não é difícil<br>[] NS/NR
	Ler a validade nesta embalagem?<br>**Ler as alternativas**	[] Muito difícil<br>[] Difícil<br>[] Não é difícil<br>[] NS/NR

Recolher as embalagens dos medicamentos disponibilizados para o entrevistado

BLOCO 5 – EMBALAGEM PRIMÁRIA – MEDICAMENTO DE VENDA SOB PRESCRIÇÃO MÉDICA

Dar a embalagem de Omeprazol ou Losec® para o entrevistado, conforme sorteio

COMPREENSÃO – EMBALAGEM PRIMÁRIA – OMEPRAZOL OU LOSEC®

O rótulo que você está vendo indica que o produto:

	É omeprazol<br>**Ler as alternativas**	[] Verdadeiro<br>[] Falso<br>[] NS/NR
	Cada cápsula contém 40 mg<br>**Ler as alternativas**	[] Verdadeiro<br>[] Falso<br>[] NS/NR

LEGIBILIDADE – EMBALAGEM PRIMÁRIA – OMEPRAZOL OU LOSEC®

O quão difícil é para você:

	Ler o nome omeprazol nesta embalagem?<br>**Ler as alternativas**	[] Muito difícil<br>[] Difícil<br>[] Não é difícil<br>[] NS/NR
	Ler os miligramas nesta embalagem?<br>**Ler as alternativas**	[] Muito difícil<br>[] Difícil<br>[] Não é difícil<br>[] NS/NR
	Ler a validade nesta embalagem?<br>**Ler as alternativas**	[] Muito difícil<br>[] Difícil<br>[] Não é difícil<br>[] NS/NR

BLOCO 6 – EMBALAGEM PRIMÁRIA (FRASCO PEQUENO)

Dar a embalagem de Dipirona ou Novalgina® para o entrevistado, conforme sorteio

COMPREENSÃO – DIPIRONA OU NOVALGINA®

O rótulo que você está vendo indica que o produto:

	Tem dipirona<br>**Ler as alternativas**	[] Verdadeiro<br>[] Falso<br>[] NS/NR
	Tem 500 mg/mL<br>**Ler as alternativas**	[] Verdadeiro<br>[] Falso<br>[] NS/NR

LEGIBILIDADE – DIPIRONA OU NOVALGINA®

O quão difícil é para você:

	Ler o nome dipirona nesta embalagem?<br>**Ler as alternativas**	[] Muito difícil<br>[] Difícil<br>[] Não é difícil<br>[] NS/NR
	Ler os miligramas nesta embalagem?<br>**Ler as alternativas**	[] Muito difícil<br>[] Difícil<br>[] Não é difícil<br>[] NS/NR
	Ler a validade nesta embalagem?<br>**Ler as alternativas**	[] Muito difícil<br>[] Difícil<br>[] Não é difícil<br>[] NS/NR
	Ler para o que serve esse remédio?<br>**Ler as alternativas**	[] Muito difícil<br>[] Difícil<br>[] Não é difícil<br>[] NS/NR

Recolher as embalagens dos medicamentos disponibilizados para o entrevistado

A partir de agora vou lhe fazer algumas perguntas sobre a sua experiência, de forma geral, com os rótulos de remédios disponíveis no Brasil.

BLOCO 7 – SATISFAÇÃO

Então, considerando sua experiência com embalagens de remédios de uma maneira geral:

	O quão difícil é para você **entender** as informações que estão escritas nas embalagens?<br>**Ler as alternativas*	[] Muito difícil<br>[] Difícil<br>[] Não é difícil<br>[] NS/NR
	Na maioria das vezes, quão difícil é para você **ler** as informações que estão escritas nas embalagens?<br>**Ler as alternativas*	[] Muito difícil<br>[] Difícil<br>[] Não é difícil<br>[] NS/NR
	Você costuma ler o endereço da empresa que produz o remédio nas embalagens?<br>**Ler as alternativas*	[] Sim<br>[] Não<br>[] NS/NR
	Você costuma ler nas embalagens quem é o responsável técnico pelo remédio?<br>**Ler as alternativas*	[] Sim<br>[] Não<br>[] NS/NR
	Você costuma ler a frase "TODO MEDICAMENTO DEVE SER MANTIDO FORA DO ALCANCE DAS CRIANÇAS"	[] Sim<br>[] Não<br>[] NS/NR
	Na sua opinião, é importante que diga na embalagem para que serve o remédio?<br>**Ler as alternativas*	[] Sim<br>[] Não<br>[] NS/NR
	Na sua opinião, é importante que diga na embalagem quem não pode tomar o remédio?<br>**Ler as alternativas*	[] Sim<br>[] Não<br>[] NS/NR
	Na sua opinião, o que poderia contribuir para melhorar as embalagens dos remédios:	
	Aumentar o tamanho de letra?	[] Sim<br>[] Não<br>[] NS/NR
	Chamar atenção para os miligramas usando cores?	[] Sim<br>[] Não<br>[] NS/NR
	Escrever a validade maior e em preto?	[] Sim<br>[] Não<br>[] NS/NR
	Ter caixas menos coloridas?	[] Sim<br>[] Não<br>[] NS/NR
	Diminuir a marca da empresa?	[] Sim

		[] Não<br>[] NS/NR
	Ter cores ou desenhos nas caixas para diferenciar para o que cada remédio serve	[] Sim<br>[] Não<br>[] NS/NR
	Numa nota de 0 a 10, quanto você está satisfeito(a) com as embalagens dos remédios disponíveis no Brasil.	

A partir de agora vou lhe mostrar algumas imagens de embalagens de remédios e vou lhe fazer algumas perguntas sobre isso.

**BLOCO 8 – PROTÓTIPOS**

	**PROTÓTIPO 1** – Olhando para estas quatro embalagens, qual o menor tamanho de letra em que você consegue ler confortavelmente as informações sobre o remédio?	[] Embalagem 1 (protótipo 1)<br>[] Embalagem 2 (protótipo 2)<br>[] Embalagem 3 (protótipo 3)<br>[] Embalagem 4 (protótipo 4)<br>[] NS/NR
	**PROTÓTIPO 2** – Olhando para estas embalagens, em qual delas é mais fácil enxergar que os miligramas do remédio são diferentes?	[] Par de Embalagens A (original)<br>[] Par de Embalagens B (protótipo)<br>[] Indiferente<br>[] NS/NR
	**PROTÓTIPO 3** – Olhando para estas duas embalagens, em qual delas é mais fácil ler as informações sobre o remédio?	[] Embalagem A (original)<br>[] Embalagem B (protótipo)<br>[] Indiferente<br>[] NS/NR
	**PROTÓTIPO 4** – Olhando para estas duas embalagens, em qual delas é mais fácil ler as informações sobre o remédio?	[] Embalagem A (original)<br>[] Embalagem B (protótipo)<br>[] Indiferente
	**PROTÓTIPO 5** – Olhando para estas duas embalagens, na sua opinião, diminuir a largura da tarja amarela torna mais difícil identificar que o remédio é genérico?	[] Sim<br>[] Não<br>[] NS/NR
	**Protótipo 5.1 -** Na sua opinião, se a frase "Medicamento genérico Lei nº9.787, de 1999" fosse tirada da embalagem, você conseguiria saber que o remédio é genérico?<br>**Colocar seta indicando a frase no protótipo 5**	[] Sim<br>[] Não<br>[] NS/NR
	**PROTÓTIPO 6** – Olhando para estas duas embalagens, em qual delas é mais fácil enxergar a data de validade do remédio?	[] Embalagem A (original)<br>[] Embalagem B (protótipo)<br>[] Indiferente<br>[] NS/NR
	**PROTÓTIPO 7** – Olhando estas embalagens, na sua opinião, em qual dos	[] Par de Embalagens A (original)<br>[] Par de Embalagens B (protótipo)

	pares é mais fácil ver que são dois remédios diferentes?	[] Indiferente<br>[] NS/NR
	**PROTÓTIPO 8**– Olhando para estas duas embalagens, em qual delas é mais fácil ler as informações sobre o remédio?	[] Embalagem A (original)<br>[] Embalagem B (protótipo)<br>[] Indiferente<br>[] NS/NR
	**PROTÓTIPO 9** – Olhando para estas duas embalagens, em qual delas é mais fácil ler o nome do remédio?	[] Embalagem A (original)<br>[] Embalagem B (protótipo)<br>[] Indiferente<br>[] NS/NR
	**PROTÓTIPO 10** – Olhando para estas embalagens, em qual delas é mais fácil ler o nome do remédio?	[] Par de Embalagens A (original)<br>[] Par de Embalagens B (protótipo)<br>[] Indiferente<br>[] NS/NR
	**PROTÓTIPO 11** – Olhando para estas duas embalagens, em qual delas é mais fácil enxergar a data de validade do remédio?	[] Embalagem A (original)<br>[] Embalagem B (protótipo)<br>[] Indiferente<br>[] NS/NR
	**PROTÓTIPO 12** – Olhando para estas embalagens, se você tivesse que tomar esse remédio, em qual delas é mais fácil visualizar as informações importantes para tomar o remédio?	[] Embalagem A (original)<br>[] Embalagem B (protótipo)<br>[] Indiferente<br>[] NS/NR

PROTÓTIPOS QUE SERÃO UTILIZADOS NAS ENTREVISTAS

PROTÓTIPO 1 – Tamanho Mínimo de Letra

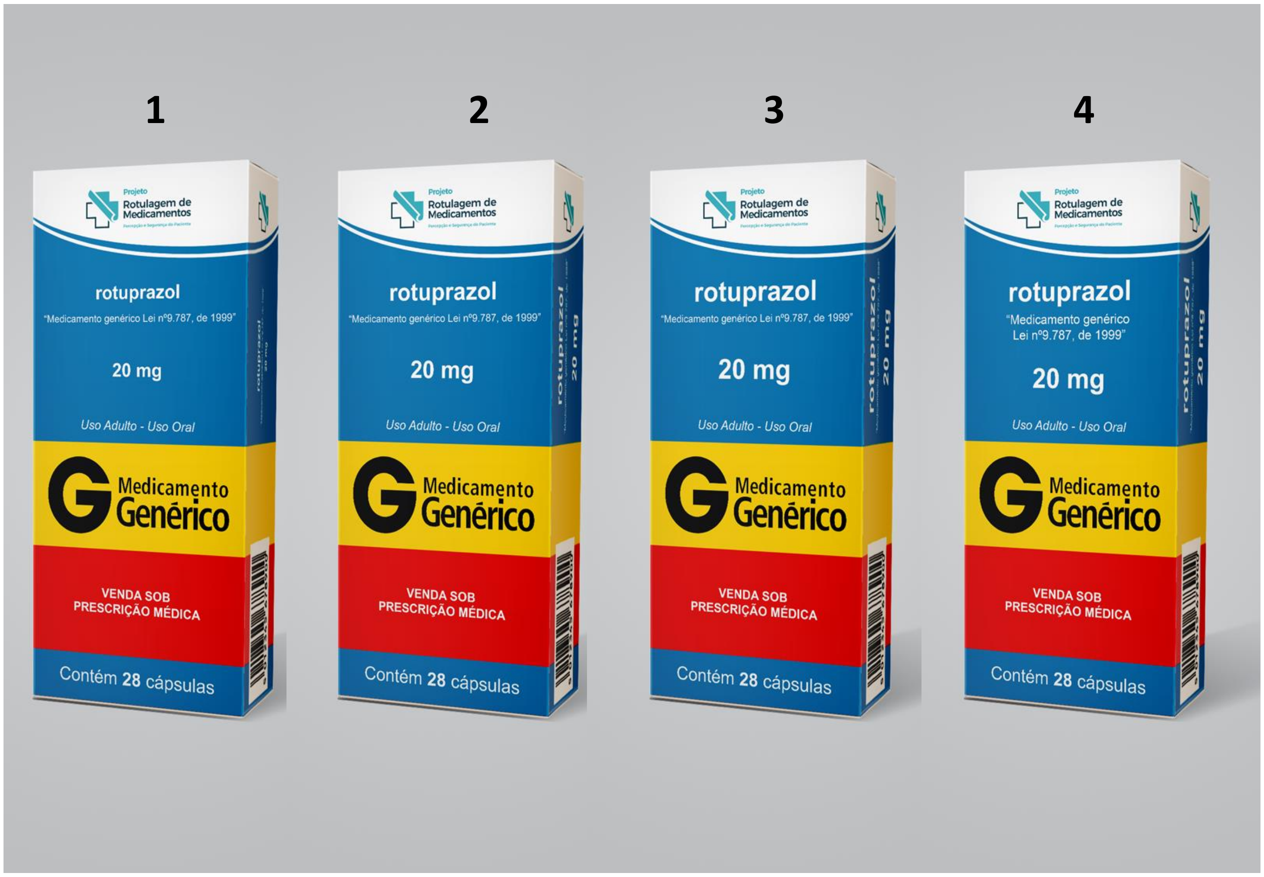

PROTÓTIPO 2 - Destaque entre Dosagens de um mesmo Medicamento

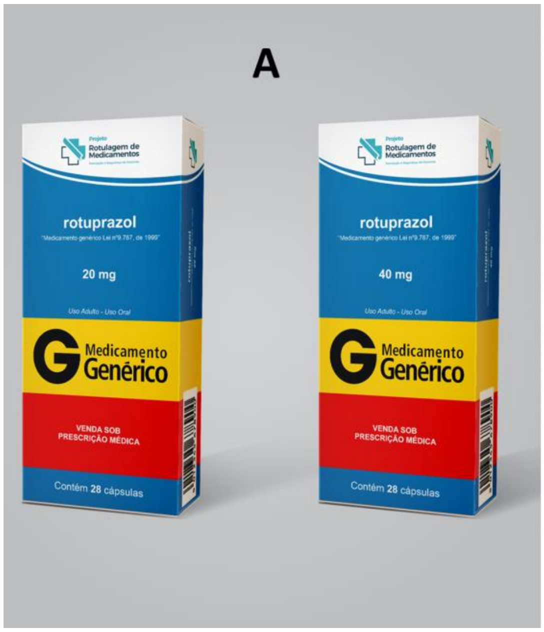

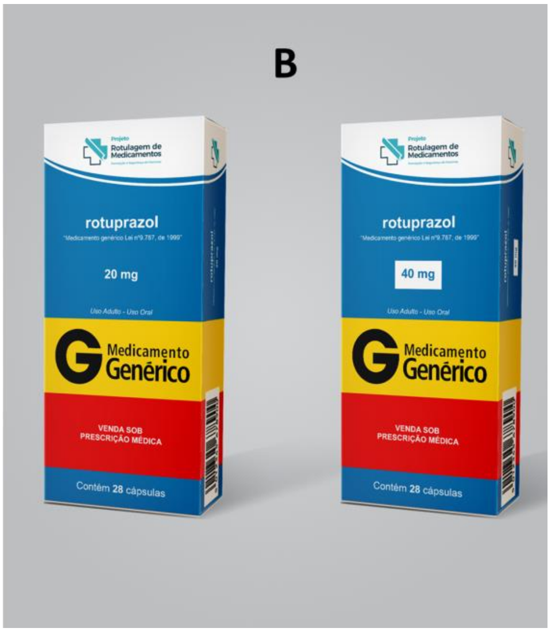

PROTÓTIPO 3 – Orientação das Informações

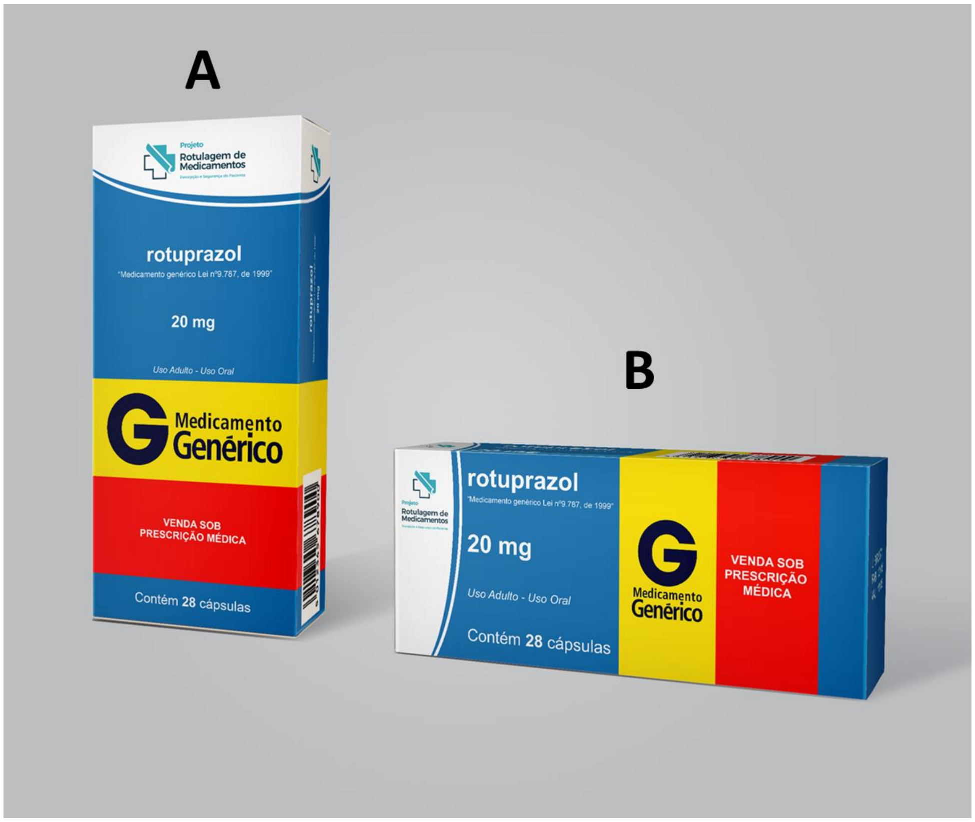

PROTÓTIPO 4 – Cores Contrastantes entre Embalagem e Informações

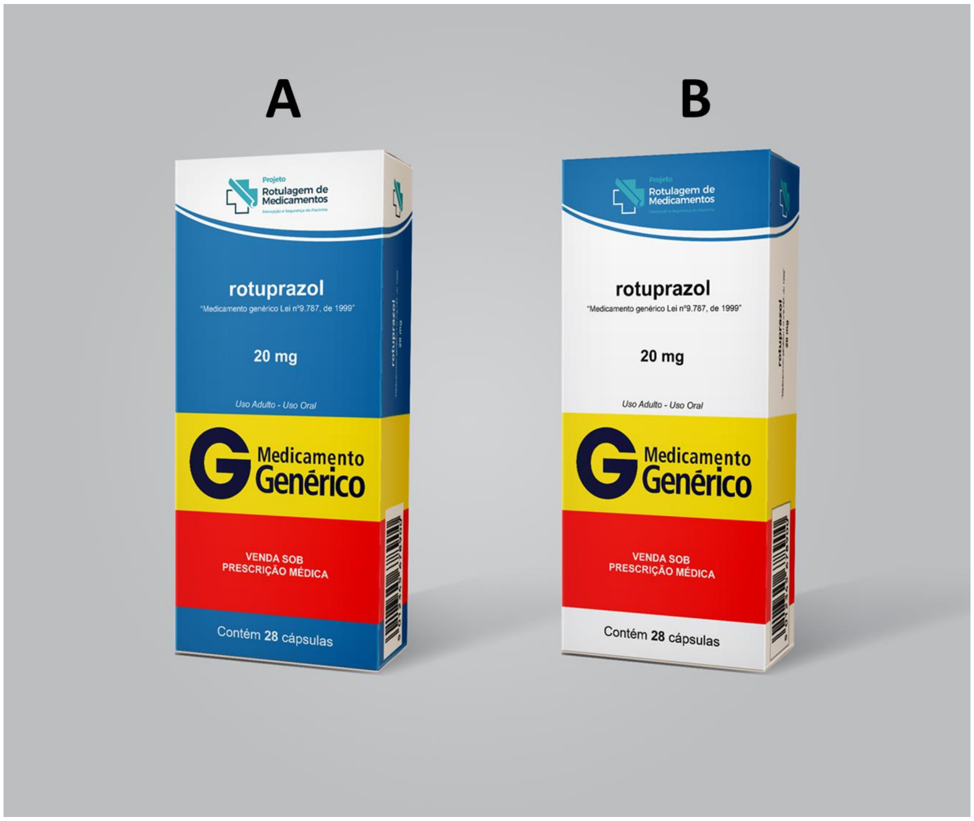

PROTÓTIPO 5 – Redução das Tarjas Amarelo e Vermelha

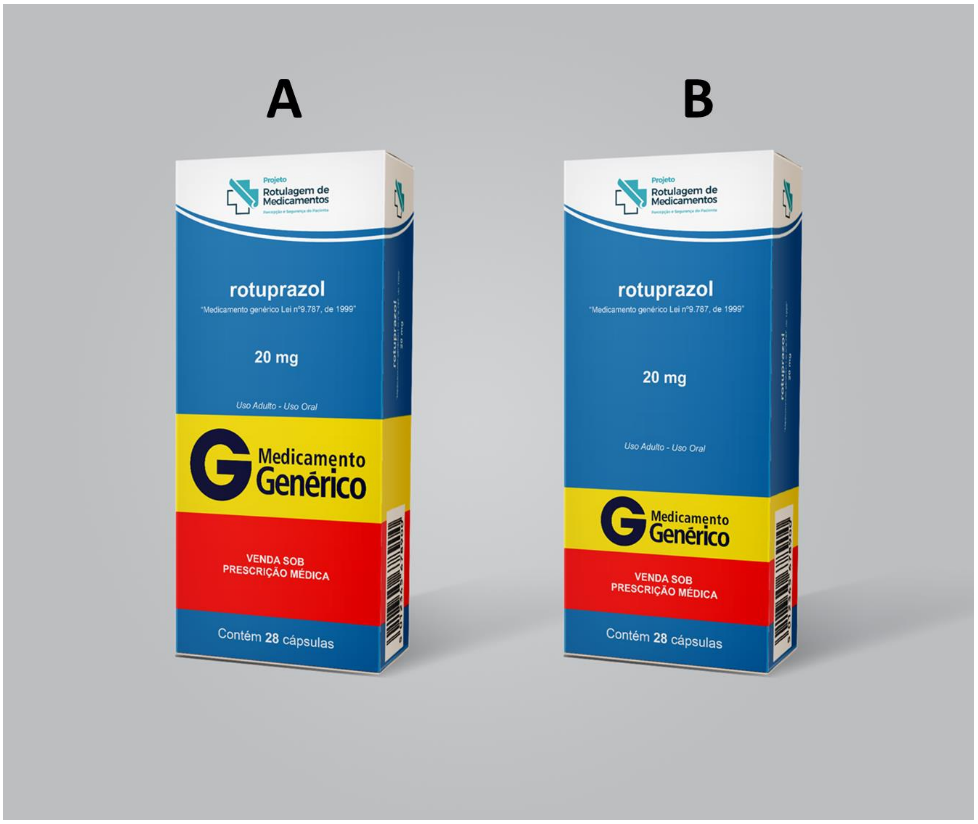

PROTÓTIPO 6 - Data de Validade em Caixas de Medicamentos

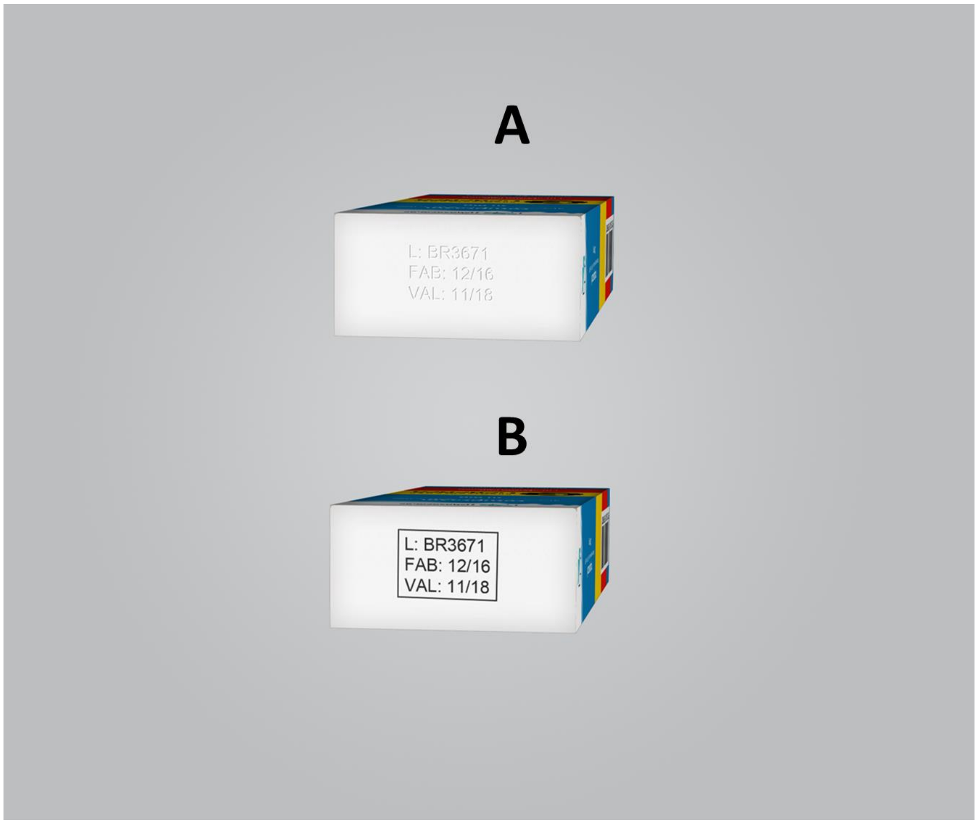

PROTÓTIPO 7 - Cores para Identificar Classe Terapêutica

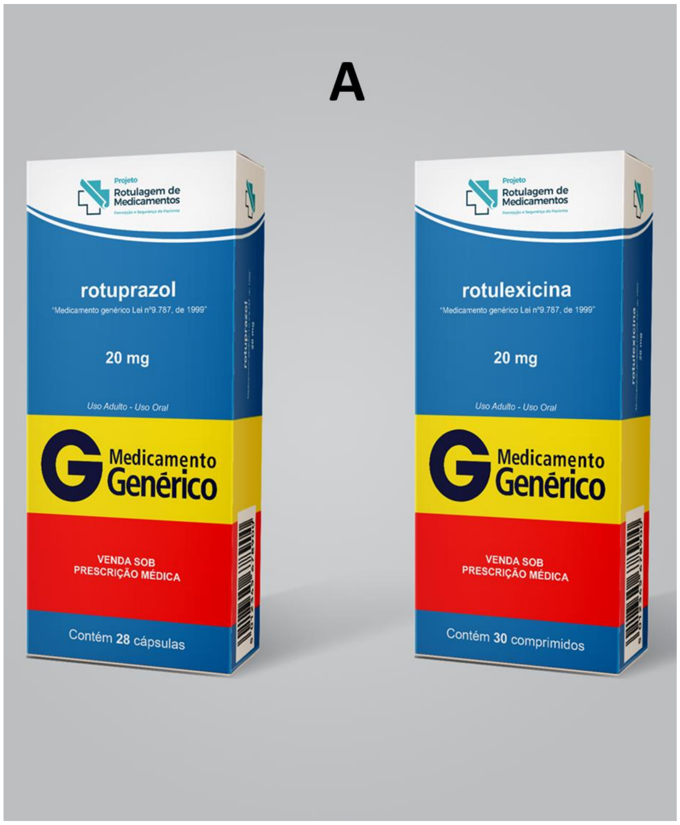

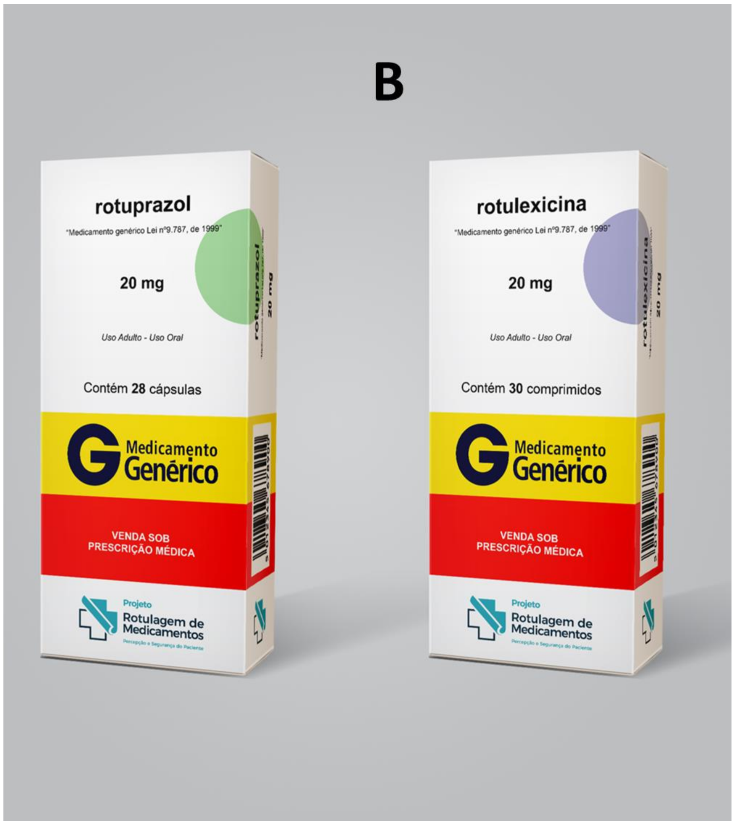

PROTÓTIPO 8 - Blíster com Cor de Fundo Contrastante

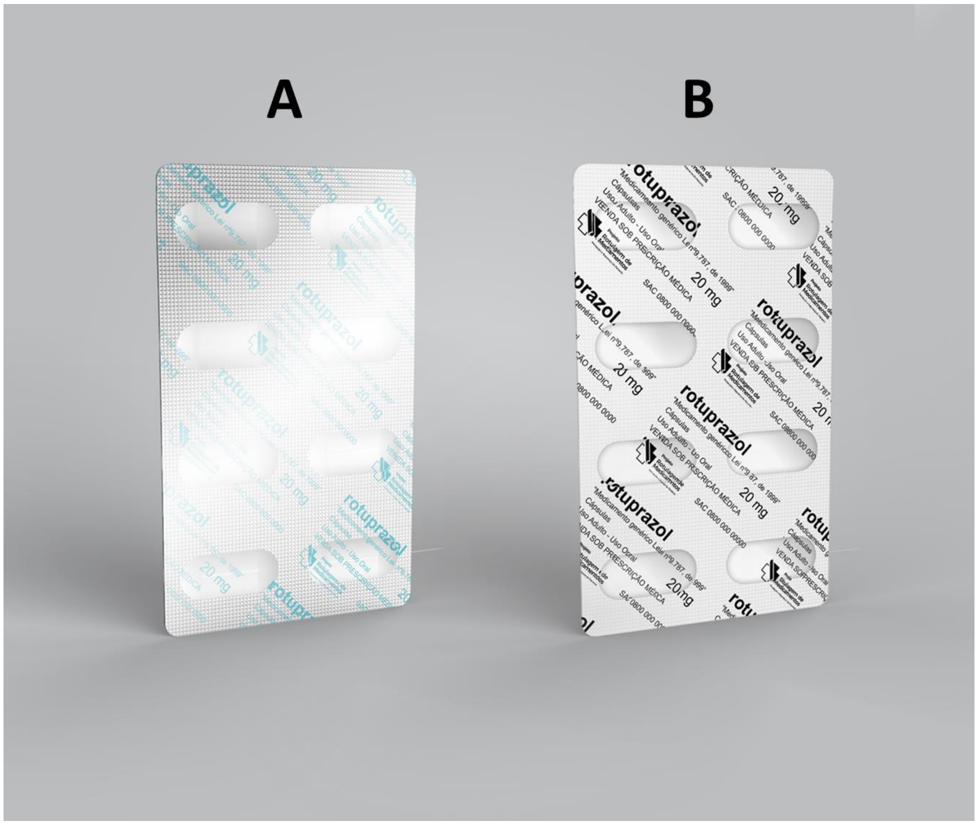

PROTÓTIPO 9 - Blíster com Dose Unitarizada para Medicamentos de Venda sob Prescrição Médica

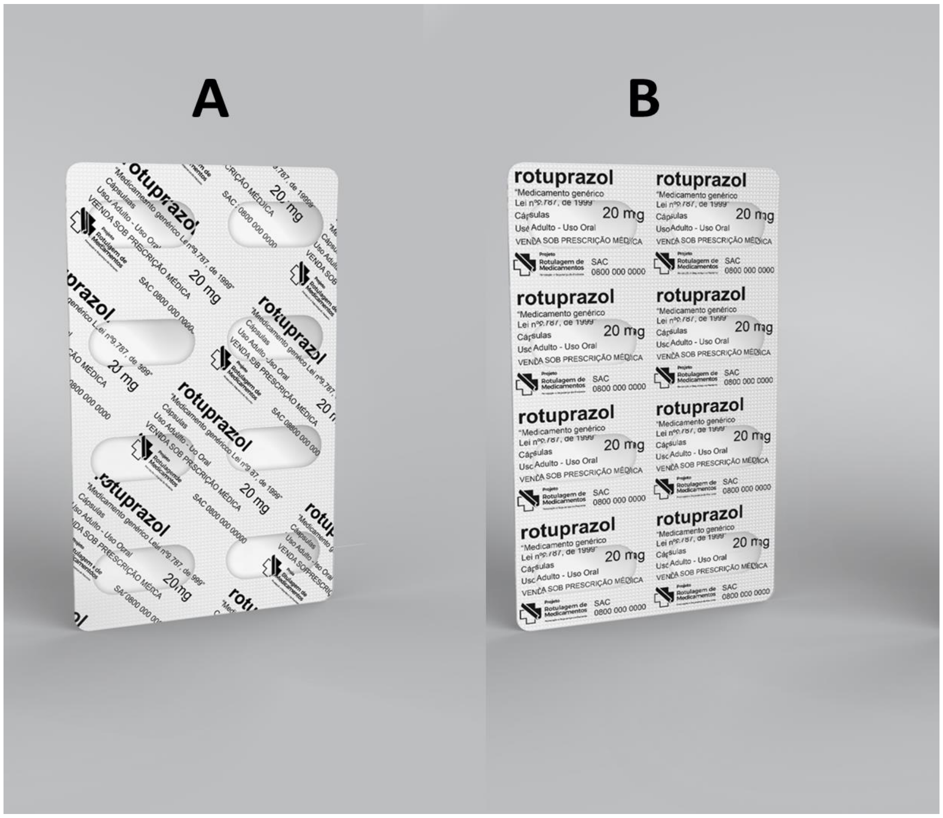

Hospital Moinhos de Vento | Afiliado a Johns Hopkins Medicine International
PROADI-SUS Programa de Apoio ao Desenvolvimento Institucional do Sistema Único de Saúde

PROTÓTIPO 10 – Blíster para Medicamentos de Venda Livre

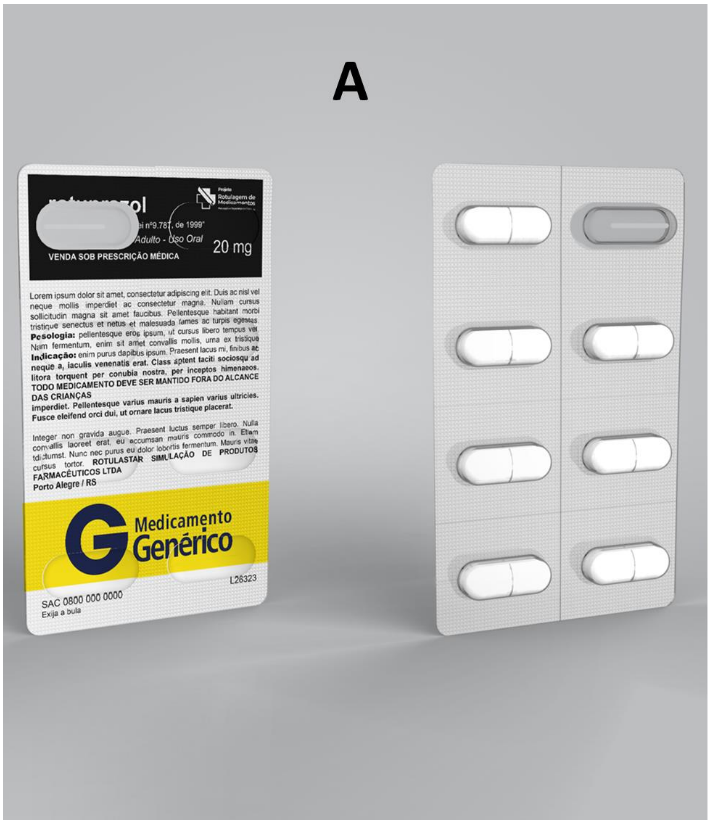

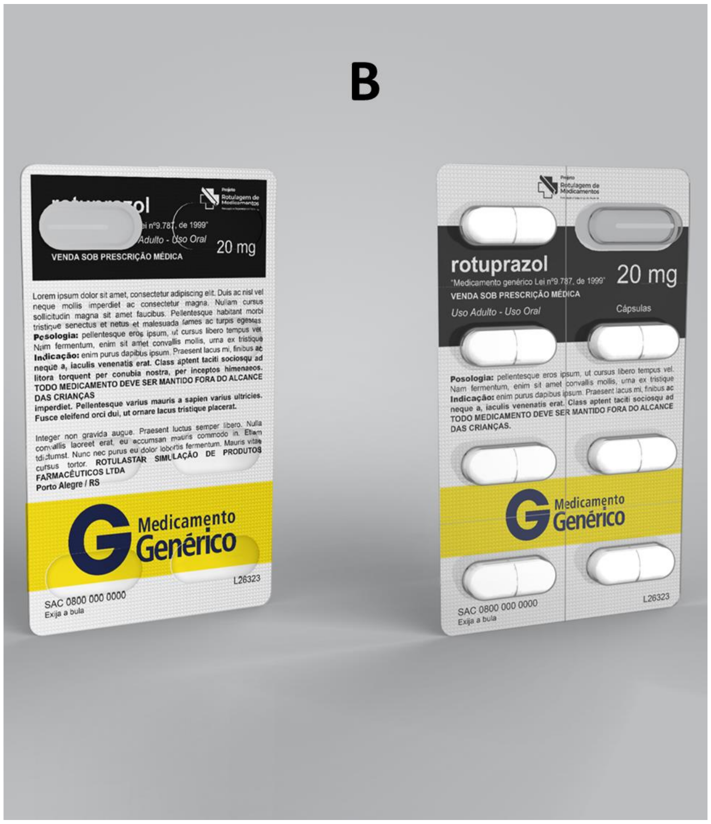

PROTÓTIPO 11 – Data de Validade em Blíster

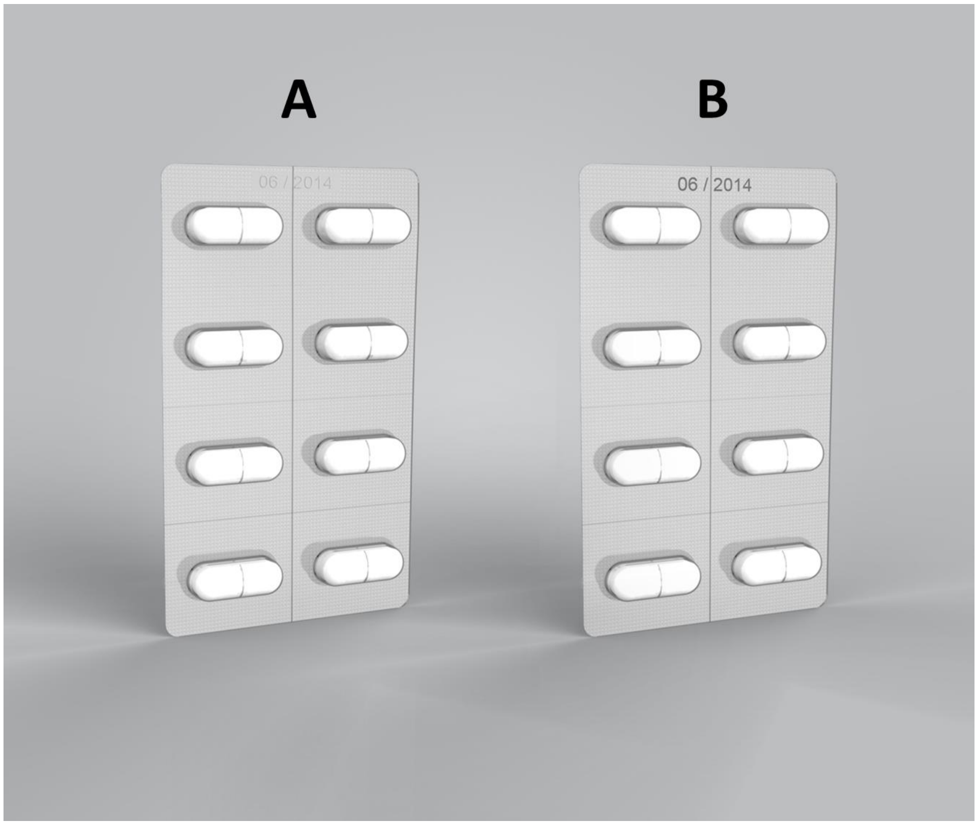

PROTÓTIPO 12 – Embalagem Ideal

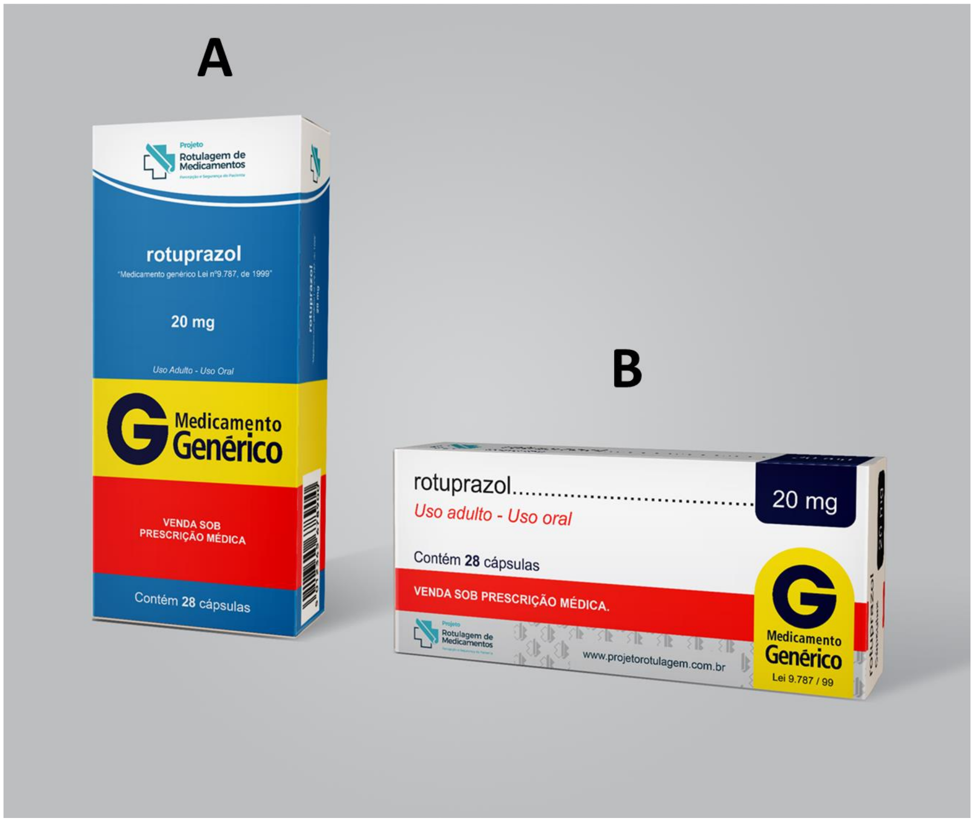

Supplement: S5 Questionnaire — (PDF) [file pone.0212173.s006.pdf]
